# Aging influences the cardiac macrophage phenotype and function during steady state and during inflammation

**DOI:** 10.1111/acel.13438

**Published:** 2021-08-02

**Authors:** Noushin Saljoughian Esfahani, Qian Wu, Naresh Kumar, Latha Prabha Ganesan, William P. Lafuse, Murugesan V. S. Rajaram

**Affiliations:** ^1^ Department of Microbial Infection and Immunity/ College of Medicine The Ohio State University Wexner Medical Center Columbus OH USA; ^2^ Department of Internal Medicine College of Medicine The Ohio State University Wexner Medical Center Columbus OH USA

**Keywords:** aging, cardiac macrophages, infection and inflammation

## Abstract

Aging‐mediated immune dysregulation affects the normal cardiac immune cell phenotypes and functions, resulting in cardiac distress. During cardiac inflammation, immune activation is critical for mounting the regenerative responses to maintain normal heart function. We investigated the impact of aging on myeloid cell phenotype and function during cardiac inflammation induced by a sub‐lethal dose of LPS. Our data show that hearts of old mice contain more myeloid cells than the hearts of young mice. However, while the number of monocytic‐derived suppressor cells did not differ between young and old mice, monocytic‐derived suppressor cells from old mice were less able to suppress T‐cell proliferation. Since cardiac resident macrophages (CRMs) are important for immune surveillance, clearance of dead cells, and tissue repair, we focused our studies on CRMs phenotype and function during steady state and LPS treatment. In the steady state, we observed significantly more MHC‐II^low^ and MHC‐II^high^ CRMs in the hearts of old mice; however, these populations were decreased in both young and aged mice upon LPS treatment and the decrease in CRM populations correlated with defects in cardiac electrical activity. Notably, mice treated with a liver X receptor (LXR) agonist showed an increase in MerTK expression in CRMs of both young and old mice, which resulted in the reversal of cardiac electrical dysfunction caused by lipopolysaccharide (LPS). We conclude that aging alters the phenotype of CRMs, which contributes to the dysregulation of cardiac electrical dysfunction during infection in aged mice.

## Graphical abstract

Self‐renewing MerTK+ cardiac resident macrophages (CRMs) is detrimental for steady‐state heart function during endotoxin (LPS) shock. Age‐associated decline in proliferation of CRMs promotes the cardiac inflammation and subsequent cardiac electrical dysfunction. LXR agonist treatment enhances the MerTK expression and MerTK+ CRM population in the hearts of old mice and provides cardio protection.

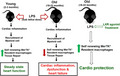

## 1 INTRODUCTION

The global geriatric population is growing and is estimated to reach about 2.1 billion by 2050, by which time this population will have longer life expectancy as well (United Nations, 2017). Heart failure increases with age (Calder et al., 2017) and accounts for most of the hospitalization of patients 65 or older (Diez‐Villanueva & Alfonso, 2016). Age‐associated increase in coronary disease, hypertension, and diabetes are contributing factors to the incidence of heart failure in the elderly. The presence of bacterial infections also increases heart failure (Demissei et al., 2016; Headley et al., 2019; Makara et al., 2016; Smit et al., 2016; Wang et al., 2014). Heart failure associated with cardiac aging is due to left ventricular hypertrophy and fibrosis, resulting in diastolic dysregulation (Costantino et al., 2016; Kovacic et al., 2011). The elderly have attenuated immune function due to senescence of the adaptive immune system, which is accompanied by low‐grade chronic inflammation termed “inflammaging” (Cevenini et al., 2013; Franceschi et al., 2000). Inflammaging is linked to increased risk of chronic diseases, such as cancer, cardiovascular diseases, infectious diseases, and premature death (Bruunsgaard & Pedersen, 2003; Pawelec et al., 2014; Singh & Newman, 2011). Cardiac aging is characterized by cardiomyocyte hypertrophy, inflammation, and the gradual development of cardiac fibrosis (Meschiari et al., 2017). In addition, the aging heart shows decreased elasticity and increased stiffness (Cieslik et al., 2011; Oh et al., 2017). Emerging evidence from studies in experimental animals and in humans has emphasized that excessive reactive oxygen species (ROS) and superoxide generated by oxidative stress and low‐grade inflammation (inflammaging) are the major causes for age‐related cardiovascular diseases (CVD) (de Almeida et al., 2020; Panth et al., 2016; Papaconstantinou, 2019; Sies, 2015; Wu et al., 2014). However, the mechanism of infection‐mediated cardiac dysfunction during aging and the role of aged cardiac immune cells is not well studied. Recently, we demonstrated that *Mycobacterium avium* infection in old mice resulted in dissemination of mycobacteria into the heart tissue and caused cardiac dysfunction (Headley et al., 2019). *M*. *avium* infected aged mice had significant premature atrial contractions and cardiac dysrhythmia, increased inflammation, and cardiac fibrosis.

Infection with pathogens can also lead to sepsis, which is characterized by excessive inflammatory response to the pathogen (van der Poll et al., 2017). Sepsis is a disease of the elderly with more than half of sepsis cases occurring in patients older than 65 years (Martin et al., 2006; Mayr et al., 2010). Sepsis caused by gram‐negative bacteria is largely due to release of lipopolysaccharide (endoxin), which triggers the inflammatory response by binding to the Toll‐like receptor TLR4. In the heart, the inflammatory response induced by sepsis causes decreased ventricular function and myocardial damage, which may progress to decreased cardiac output and death (Parker et al., 1984). While studies have defined the damage to the heart caused by severe sepsis, less is known about the damage to the heart caused by low‐level inflammation. Thus, in the current study, we investigated the effect of aging on the composition of myeloid cells in the heart during steady state and after an inflammatory event induced by a sub‐lethal dose of LPS.

The heart is immunologically active even during normal functioning and contains all major leukocyte populations. The majority of leukocytes present in the steady‐state functioning adult mice heart are F4/80^+^ CD11b^+^ macrophages, whereas other immune cells, including mast cells, dendritic cells (DCs), B cells, and regulatory T cells, are found minimally in cardiac tissue (Epelman et al., 2014, 2015; Ramos et al., 2017). Monocytes and neutrophils are not normally seen in myocardial tissue of the adult steady‐state heart, but are seen during an inflammatory stage (Epelman et al., 2014, 2015; Ramos et al., 2017). Macrophage numbers in the heart of mice have been reported to either decrease or increase with age. A study by Ramos et al. (2017) showed that mice aged 12–15 months have decreased numbers of monocytes/macrophages and increased numbers of neutrophils. Other studies (Ma et al., 2018; Salminen et al., 2019) have reported that cardiac macrophages increase in number beginning at 18 months of age and that the number positively correlates with age.

The mouse heart at steady state contains macrophages subsets with distinct functions and origins. The cardiac macrophages can be grouped into three subsets based on their origin and their expression of CC chemokine receptor 2 (CCR2) and MHC class II. The two CCR2^−^ macrophages subsets (CCR2^−^ MHC‐II^high^, CCR2^−^ MHC‐II^low^) are of embryonic origin and maintain their numbers through self‐renewal rather than through infiltration of blood monocytes. The third subset is CCR2^+^ MHC‐II^high^ macrophages, which are maintained by blood monocyte recruitment. Transcriptional profile analysis showed that the CCR2^+^ macrophages are enriched for genes involved in inflammatory pathways, suggesting that these macrophages are inflammatory in nature (Lafuse et al., 2020). All CRM subsets were efficient in taking up dead cardiomyocytes, indicating the critical role of CRMs in clearing dead cells and cell debris. The cells that most efficiently engulfed dead cells in the cardiac environment were the CCR2^−^ MHC‐II^low^ macrophages, which can clear dead cells without triggering an immune response (Epelman et al., 2014). Also, MerTK, a phagocytic receptor involved in phagocytosis of apoptotic cells, is highly expressed in cardiac resident macrophages (Ma et al., 2018; Salminen et al., 2019). Thus, each of the CRM subsets has a distinct role in the response to cardiac injury, triggering an inflammatory response, clearing dead cells and debris, or acting as antigen‐presenting cells. However, the cardiac immune cell phenotypic changes during infection in aged mice are not known.

In the current study, we examined the effect of aging and inflammation on the phenotypic changes of myeloid cells and macrophage functions in the heart. We observed that aging increased the number of neutrophils and monocytes in steady‐state hearts, but did not significantly increase total macrophage numbers. After low‐dose LPS injection of mice, the numbers of total CRMs, MHC‐II^high^ CRMs, and MHC‐II^low^ CRMs were significantly reduced in both young and old mice. CRMs expressing MerTK were also reduced in young and old mice. Further, we report that low‐dose LPS significantly altered cardiac electrical activity in both young and old mice; however, the effect of LPS was more prominent in old mice. We also report that treatment of bone marrow‐derived macrophages with a LXR agonist increases MerTK expression, and *in vivo* treatment with the LXR agonist increased MerTK expression in all three CRM populations in both young and old mice. Furthermore, we found that the LXR treatment reversed the LPS‐induced cardiac electrical dysfunction in old mice.

## 2 RESULTS

### 2.1 Aging enhances the accumulation of myeloid cells in the heart

**FIGURE 1:**
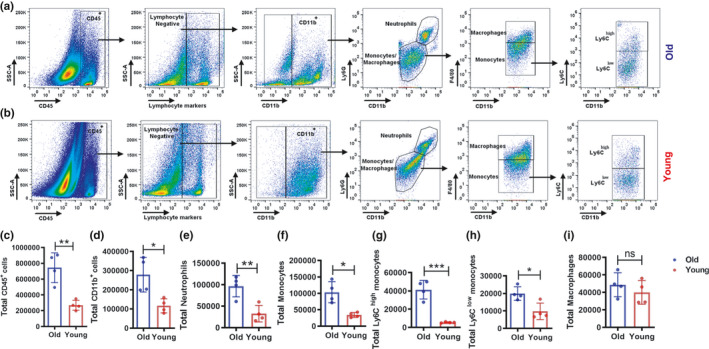
Phenotypic analysis of myeloid cells in the hearts of young and old mice. The myeloid cell abundance in the heart of old and young was examined by flow cytometry. Old (18 months) and young (6–8 weeks) C57BL/6 mice were euthanized and hearts digested to obtain single‐cell suspensions. Cells were labeled with different myeloid cells markers and analyzed by flow cytometry. Representative flow cytometry plots and gating strategy are shown for heart myeloid cells from old (a) and young (b) mice. The total number of cells per heart from old and young mice was determined for (c) leukocytes (CD45^+^), (d) myeloid cells (CD11b^+^), (e) neutrophils (CD11b^+^Ly6G^+^), (f) monocytes (CD11b^+^F4/80^−^), (g and h) Ly6C high and low monocytes (CD11b^+^F4/80^−^Ly6C^high/low^), and (i) macrophages (CD11b^+^F4/80^+^). Data shown in the graphs are from a representative experiment of three independent experiments (4 mice per group). A two‐tailed Student's *t* test was used to analyze the data (*p < 0.05 and ** *p *< 0.005)

Myeloid immune cells play a critical role in steady‐state heart function, immune surveillance, cardiac inflammation, and fibrosis (Epelman et al., 2014, 2015; Ramos et al., 2017). Aging‐mediated chronic inflammation is associated with many diseases, including cardiovascular diseases (Bruunsgaard & Pedersen, 2003; Costantino et al., 2016; Pawelec et al., 2014; Singh & Newman, 2011). Therefore, we examined whether aging alters the myeloid cell population in steady‐state hearts. We harvested hearts from young (6–8 weeks old) and old (18 months old) C57BL/6 mice. Single‐cell suspensions were prepared by enzymatic digestion (Meeson et al., 2013) and analyzed by flow cytometry. The gating strategy and representative flow cytometry plots are shown in Figure 1 for old mice (a) and young mice (b). The fluorescence minus ones (FMO’s) for all myeloid cell markers are shown in Figure S1. We identified leukocytes, myeloid cells, neutrophils, monocytes (Ly6C^high^ and Ly6C^low^), and macrophages. Total leukocytes (CD45^+^ cells) were considerably more prevalent in the hearts of old mice compared with those of young mice (Figure 1c). This finding is consistent with our previously reported immunohistochemistry data, which showed a larger CD45^+^ leukocyte population in the cardiac muscle of old mice compared with that of young mice (Headley et al., 2019). Furthermore, lymphocyte‐negative CD11b^+^ myeloid cells were significantly more prevalent in the hearts of old mice compared with those of young mice (Figure 1d). To determine the specific phenotypes of myeloid cells that contribute to the difference in CD11b^+^ cell numbers, we analyzed the number of neutrophils (Ly6G^+^), monocytes (Ly6C^+^), and macrophages (F4/80^+^) in heart tissue of old and young mice. Among the CD11b^+^ myeloid cells, neutrophils and both Ly6C^high/low^ monocyte subsets were significantly more prevalent in old mice compared with young mice (Figure 1e–h). However, there were no significant differences in the total CRM population (Figure 1i) in the steady‐state hearts of old and young mice. Macrophages efferocytose apoptotic cells via phosphatidylserine receptors, such as MerTK. Thus, we determined the mRNA expression of MerTK in the CRMs isolated from the hearts of young and old mice. Interestingly, we found that MerTK mRNA expression in CRMs of old mice at steady state was slightly lower than in young mice (Figure S2). Thus, we conclude that hearts of old mice contain a significantly increased number of myeloid cells, which suggests that aging‐mediated chronic inflammation persists in the heart tissue.

### 2.2 Aging affects myeloid‐derived suppressor cell populations and their ability to suppress T‐cell proliferation

**FIGURE 2:**
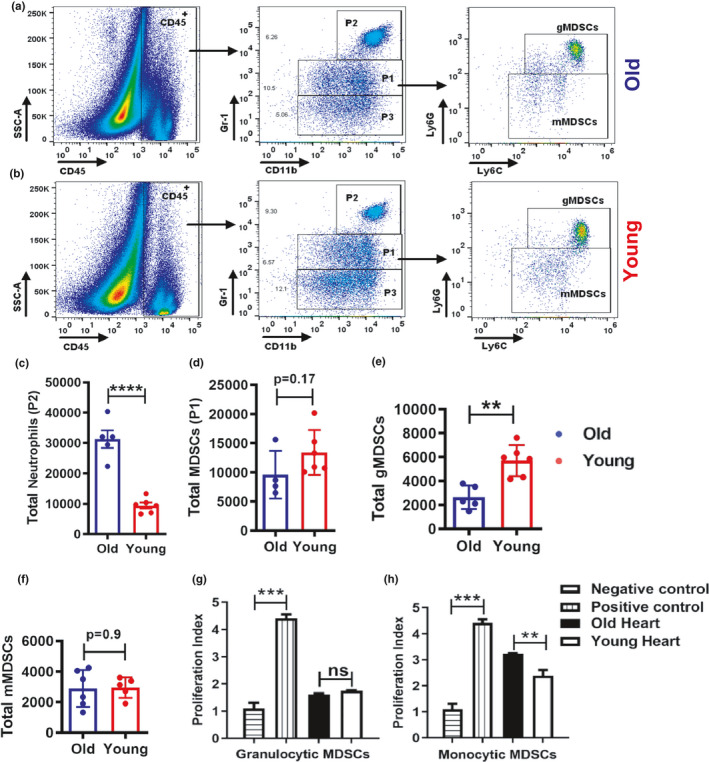
Quantification of monocyte‐derived suppressor cells in the hearts of young and old mice. Old mice contain fewer monocytic MDSCs in the heart than young mice and are less efficient in suppressing T‐cell proliferation in vitro. Heart single‐cell suspensions from old and young mice were labeled with antibodies specific for myeloid‐derived suppressor cells (MDSCs) and analyzed by flow cytometry. (a and b) representative gating strategies for heart myeloid‐derived suppressor cells in the heart of old and young mice, respectively. The graphs show the total number of cells per heart from old and young mice. (c) Neutrophils (CD11b^+^Gr1^high^‐P2), (d) Total MDSCs (CD11b^+^Gr1^int+^‐P1), (e) Granulocytic MDSCs (CD11b^+^Gr1^int^ Ly6G^+^Ly6C^+^), and (f) Monocytic‐MDSCs (CD11b^+^Gr1^low^Ly6G^−^Ly6C^+^). Monocytic and granulocytic MDSCs were sorted from heart single‐cell suspensions of old and young C57BL/6 mice and co‐cultured ex vivo with Naïve CD4^+^ T cells isolated from the spleens of young C57BL/6 mice and in vitro stimulated with anti‐CD3/CD28. (g and h) T‐cell proliferation index in the presence of granulocytic MDSCs and monocytic MDSCs, respectively, from the heart of old and young mice. The data shown in the graphs are cumulative from two independent experiments (3 mice per group). A two‐tailed Student's *t* test was used to analyze the data (*p < 0.05 and **p < 0.005)

Since myeloid‐derived suppressor cells (MDSCs) function as a regulators of inflammation and T‐cell activation (Salminen et al., 2019), we examined the MDSC population in the hearts of young and old mice during steady state. We quantified the monocytic (mMDSCs) and granulocytic (gMDSCs) subsets of MDSCs by CD11b and GR1 expression levels. GR1 binds to both the Ly6C and Ly6G antigens, but at an epitope distinct from the epitopes recognized by the Ly6C and Ly6G antibodies. Plots of CD11b and GR1 expression identified three populations (P1, P2, and P3) in both old (Figure 2a) and young (Figure 2b) mice, as defined by Greifenberg et al. (2009), Ryzhov et al. (2011) and Hüsecken et al. (2017). The P2 population consists of CD11b^high^ GR1^high^ neutrophil population, which were more prevalent in old mice than young mice (Figure 2c) and is consistent with results from the FACS analysis in Figure 1e. The P3 population is CD11b^high^ GR1^low^ monocytes. The P1 populations consist of MDSCs and are separated into gMDSCs (CD11b^high^ GR1^interm^ Ly6G^high^ Ly6C^low^) and mMDSCs (CD11b^high^ GR1^interm^ Ly6G^neg^ Ly6C^high^). Total MDSC and gMDSC populations were higher in young mice than in old mice but only the gMDSC population reached the level of statistical significance (Figure 2d,e). There was no difference in the mMDSC populations. To determine whether MDSCs isolated from the steady‐state heart exert a suppressive effect on the immune response, we co‐cultured monocytic and granulocytic MDSCs from young and old mice with carboxyfluoresceinsuccinimidyl ester‐labeled naïve CD4^+^ T cells from young mice at a ratio of 1:2 in the presence of anti‐CD3/anti‐CD28 antibodies. Even though the gMDSC population is increased in the hearts of young mice, the gMDSCs both young and old mice equally suppressed T‐cell proliferation (Figure 2g). However, mMDSCs from old mice were significantly less effective at suppressing T‐cell proliferation than mMDSCs from young mice (Figure 2h). These studies suggest that while the numbers of mMDSCs in the hearts of old mice at steady‐state do not differ from young mice, mMDSCs from old mice are deficient in suppressing T‐cell proliferation, which may contribute to chronic inflammation.

### 2.3 MerTK‐positive CRM population is decreased in the hearts of old and young mice upon endotoxin (LPS) treatment

**FIGURE 3:**
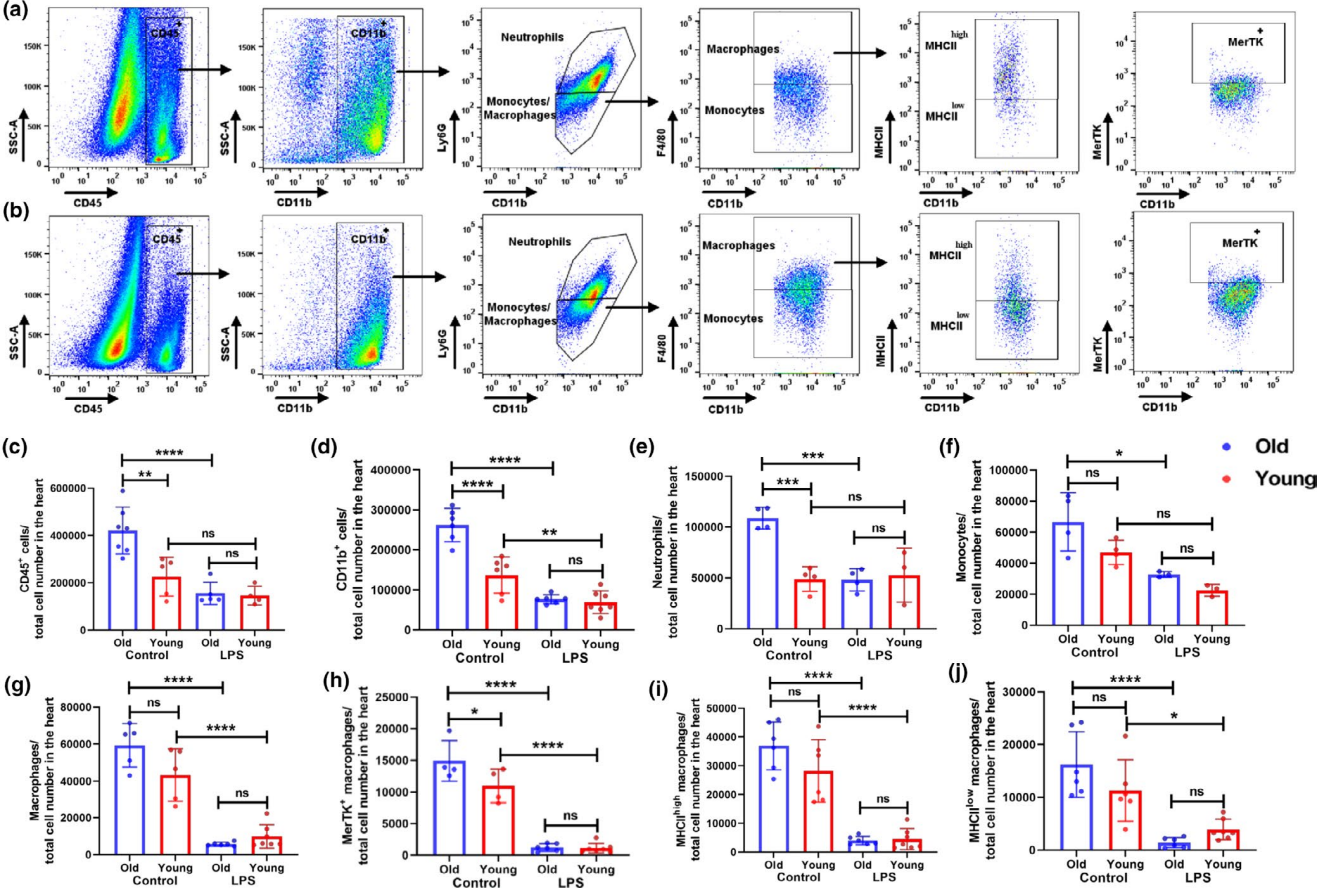
LPS infusion decreases myeloid cell expansion in the heart. Heart single‐cell suspensions from old and young mice injected with LPS (2.5 mg/kg) or mock treated for 48 h and were analyzed by flow cytometry. The cardiac immune cell populations were calculated using the same gating strategies as in Figure 1. Representative flow cytometry plots showing the gating strategies for heart macrophages from old and young mice, respectively (a and b). Data are expressed as total cells per heart: (c) CD45^+^cells, (d) CD11b^+^ myeloid cells, (e) neutrophils (CD11b^+^Ly6G^+^), (f) monocytes (CD11b^+^F4/80^−^Ly6C^+^), (g) macrophages (CD11b^+^F4/80^+^), (h). MerTK^+^ macrophages (CD11b^+^F4/80^+^MerTK^+^), and (I and J) MHC‐II high and low macrophages (CD11b^+^F4/80^+^MHC‐II^high/low^). The data shown in the graphs are cumulative from two independent experiments (3 mice per group). A one‐way ANOVA followed by the Bonferroni test was used to analyze the data (*p < 0.05 and **p < 0.005)

Myocardial dysfunction is a common complication of severe sepsis and increased mortality in ICU patients (Deutschman & Tracey, 2014; Romero‐Bermejo et al., 2011). The intraperitoneal injection of LPS has been extensively used to model many of the clinical features of sepsis, including hyper‐inflammation and cardiac dysfunction (Drosatos et al., 2013; Fallach et al., 2010). To test whether low‐dose LPS treatment alters the cardiac myeloid cell phenotypes, we infused a sub‐lethal dose of LPS (2.5 mg/kg) via intraperitoneal injection and examined the cardiac immune cell phenotypes (flow cytometry) and monitored the cardiac electrical activity (ECG) of young and old mice. We followed the gating strategy used in Figure 1 for identification of CD45 cells, neutrophils, and monocytes. The gating strategy and representative flow cytometry plots are shown in Figure 3 for old mice (a) and young mice (b). CRMs were identified as CD45^+^ CD11b^+^, Ly6G^−^, F4/80^+^ using the gating strategy shown in Figure 1. In steady‐state control mice, we observed an increased number of CD45, CD11b, neutrophils, and monocytes in old mice compared with young mice. Upon LPS stimulation, we found significant decreases in the numbers of CD45^+^ cells, CD11b^+^cells, neutrophils, and monocytes in the hearts of old mice; however, this reduction in myeloid cell in young mice was only statistically for CD11b^+^ cells (Figure 3c–f). Notably, in steady‐state control mice, there were no statistical differences between young and old mice in numbers of total CRMs, the numbers of MHC‐II^high^ and MHC‐II^low^ macrophages (Figure 3g–j). Upon LPS infusion, our data show that the total CRM population is decreased in both young and old mice (Figure 3g). Furthermore, we quantified the MerTKexpressing CRMs in young and old mice after LPS treatment. Interestingly, we found a significant reduction in the number of MerTK^+^ CRMs in the hearts of LPS‐infused young and old mice (Figure 3h). Similarly, MHC‐II high and low macrophage populations are significantly reduced in both young and old mice by LPS treatment; however, our data indicate that the numbers of MHC‐II^low^ CRM population are slightly higher in young mice upon LPS treatment (Figure 3i,j).

### 2.4 LPS treatment causes cardiac electrical dysfunction

**FIGURE 4:**
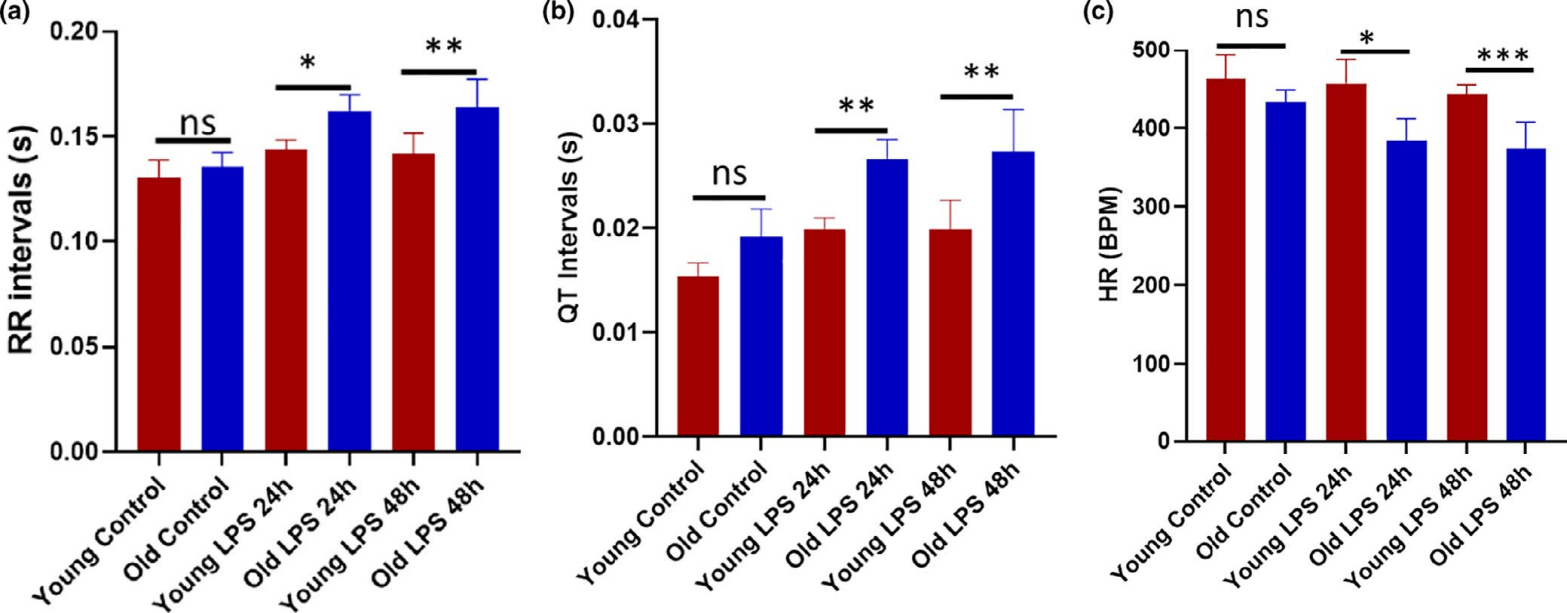
Aging augments the LPS‐mediated cardiac electrical dysfunction. Surface electrocardiogram (ECG) recordings were obtained from old and young mice injected with LPS (2.5 mg/kg) or mock‐treated control old and young mice at baseline and at 24 and 48 h post‐treatment by Powerlab 4/30 (AD Instruments). ECG traces were analyzed using LabChart 8 Pro (AD Instruments). Shown are accumulative graphs of RR intervals (a), QT intervals (b), and heart rate (c) of young and old mice (*N* = 8). A one‐way ANOVA followed by the Bonferroni test was used to analyze the data (*p < 0.05; **p < 0.005; and ***p < 0.0005)

Since we found that a significant loss of CRMs in low‐dose LPS‐infused young and old mice, we then examined the cardiac electrical activity in LPS‐injected young and old mice. We found that LPS injection significantly altered the cardiac electrical activity. LPS increased the RR and QT intervals and decreased the heart rate in both young and old mice (Figure 4a–c). Also, we found variability in RR intervals and in heart rate among individual mice. However, the effect of LPS on cardiac electrical dysfunction was more prominent (increased RR, QT intervals, and decreased heart rate) in old mice, which indicates that these mice were experiencing the cardiac arrhythmia. The graph in Figure S3 shows the variability in RR and QT intervals from representative mice. Together the data suggest that LPS‐induced cardiac electrical dysfunction may be due to the decreased CRMs in the heart tissue.

### 2.5 LXR agonist treatment enhances MerTK expression in macrophages in vitro and in vivo

Given our observation that cardiac macrophages from LPS‐infused (Figure 3) mice have reduced numbers of MerTK‐positive CRMs, and given the important role of MerTK in clearance of apoptotic cells (Lemke & Rothlin, 2008; Penberthy & Ravichandran, 2016; Wan et al., 2013), we investigated a possible mechanism to enhance the expression of MerTK in CRMs. Recently, it was shown that MerTK expression can be enhanced by LXR activation or peroxisome proliferator‐activated receptor (PPAR)‐γ inhibition in human macrophages (Zizzo & Cohen, 2015). To confirm whether MerTK in mice is also regulated by LXR activation or PPAR‐γ inhibition, we treated BMDMs from young mice with the PPAR‐γ antagonist (GW9662‐10 μM) or the LXR agonist (T0901317‐1 μM) for 24 h. MerTK mRNA levels were determined by qRT‐PCR and protein levels by Western blotting. Our results show that the treatment of BMDMs with LXR agonist (T0901317) increased MerTK mRNA compared to DMSO control treatment, in contrast, we found that the PPAR‐γ antagonist (GW9662) treatment did not increase the expression of MerTK (Figure S4a). In agreement with the qRT‐PCR data, MerTK protein expression levels were higher after treatment with the LXR agonist (T0901317) but not with the PPAR‐γ antagonist, as shown in Figure S4b,c. These results provide evidence that treatment with LXR agonist (T0901317) induces MerTK expression in mouse macrophages, but PPAR‐γ is not involved. Based on these in vitro observations, we next determined whether treatment of mice with the LXR agonist increases MerTK in vivo.

**FIGURE 5:**
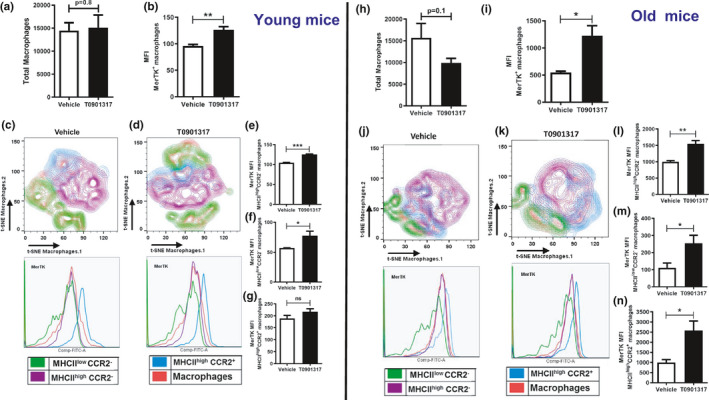
LXR agonist treatment induces the MerTK expression on the surface of MHC‐II^high/low^ macrophages in the heart of both young and old mice in vivo. Young and old female C57BL/6 mice were treated daily with either T0901317 (50 mg/kg/day) or vehicle for 9 days by intraperitoneal injections. At day 10, mice were euthanized and single cell heart expression suspensions analyzed by flow cytometry. (a and h), Number of macrophages (CD45^+^CD11b^+^Ly6G^–^F4/80^+^) per heart tissue of Vehicle and LXR agonist (T0901317) treated young and old mice, respectively. **(**b and i), Mean fluorescence intensity (MFI) of MerTK expression on cardiac macrophages after vehicle or LXR agonist (T0901317) treatment in young and old mice, respectively. (c and d) and (j and k), t‐SNE flow cytometry analysis of MerTK expression on different CRM subsets (MHC‐II^low^CCR2^−^, MHC‐II^high^CCR2^−^, and MHC‐II^high^CCR2^+^) in young and old mice, respectively. Cells were pre‐gated on CD45^+^CD11b^+^Ly6G^–^F4/80^+^ cells and subjected to unbiased t‐SNE analysis. Overlays were generated from Vehicle (c and j) or LXR agonist (T0901317) (d and k) treated young and old animals, and the clusters were then analyzed in detail for their MerTK expression profiles, as shown in the subsequent histogram overlays. (e and g) and (l–n), The graphs show the mean fluorescence intensity (MFI) of MerTK expression of each macrophages subset after vehicle or LXR agonist (T0901317) treatment in young and old mice, respectively. (e and l), MHC‐II^high^CCR2^−^, (f and m), MHC‐II^low^CCR2^−^, (g and n), MHC‐II^high^CCR2^+^. The data shown in the graphs are cumulative from three different independent experiments. A two‐tailed Student's *t* test was used to analyze the data (**p < 0.005 and ***p < 0.001)

We treated young and old mice with vehicle (10% Cremaphore in saline) or LXR agonist (T0901317; 50 mg/kg/day [Gao & Liu, 2013]) for 9 days via daily intraperitoneal injection. We then determined whether there were cardiac macrophage expansion as well as increased MerTK expression (Figure 5). Data from flow cytometry analysis revealed that, although LXR agonist did not expand the CRM population in young or old mice (Figures 5a and 5h), there was greater expression of MerTK on CRMs in mice treated with LXR agonist compared with vehicle‐treated young and old mice (Figure 5b and 5i). Next, to determine whether the LXR agonist‐mediated induction of MerTK occurred in specific subsets of CRMs, the different subsets of CRMs, including CRMs of embryonic origin (MHC‐II^low^ CCR2^−^ and MHC‐II^high^ CCR2^−^) and monocyte‐derived CRMs (MHC‐II^high^ CCR2^+^), were characterized using multicolor flow cytometry analysis and an unbiased t‐SNE algorithm analysis of CD45^+^CD11b^+^Ly6G^–^F4/80^+^ cell populations derived from the hearts of both vehicle and T0901317‐treated young and old mice (Figure 5c–g [young mice] and Figure 5j–n [old mice]). Although the total CRM population was not differentially expanded in vehicle and T0901317‐treated mice (Figures 5a and 5h), the expansion of embryonic derived MHC‐II^low^ CCR2^−^ and MHC‐II^high^ CCR2^−^ cardiac macrophages was higher in T0901317‐treated mice, as shown in the green and purple clusters in Figure 5d and 5k. In agreement with the induction of MerTK expression on the total CRMs observed after LXR agonist treatment, the MHC‐II^low^ CCR2^−^ and MHC‐II^high^ CCR2^−^ CRM populations had significantly higher expression of MerTK after treatment with T0901317 compared with vehicle, as shown in MFI bar graphs (Figures 5e,f and 5l,m). Notably, LXR treatment did not increase MerTK expression in monocyte‐derived cardiac macrophages (MHC‐II^high^ CCR2^+^) of young mice (Figure 5g); whereas in old mice, MerTK expression was significantly higher in the MHC‐II^high^ CCR2^+^ macrophage population (Figure 5n). Overall, LXR agonist treatment enhanced expression of MerTK in the hearts of both young and old mice; however, we found that the LXR agonist‐mediated enhancement of MerTK expression (MFI) was higher in old mice compared with young mice (Figure S5). Together, these results strongly indicate that treatment with LXR agonist can increase the expression of MerTK and enhance the MerTK‐positive macrophage populations in the hearts of both young and old mice, thereby providing a potential target for treating cardiac inflammation in old age.

### 2.6 LXR agonist treatment enhances the accumulation of MerTK‐positive macrophages and reverses the cardiac electrical dysfunction caused by LPS in the mice

**FIGURE 6:**
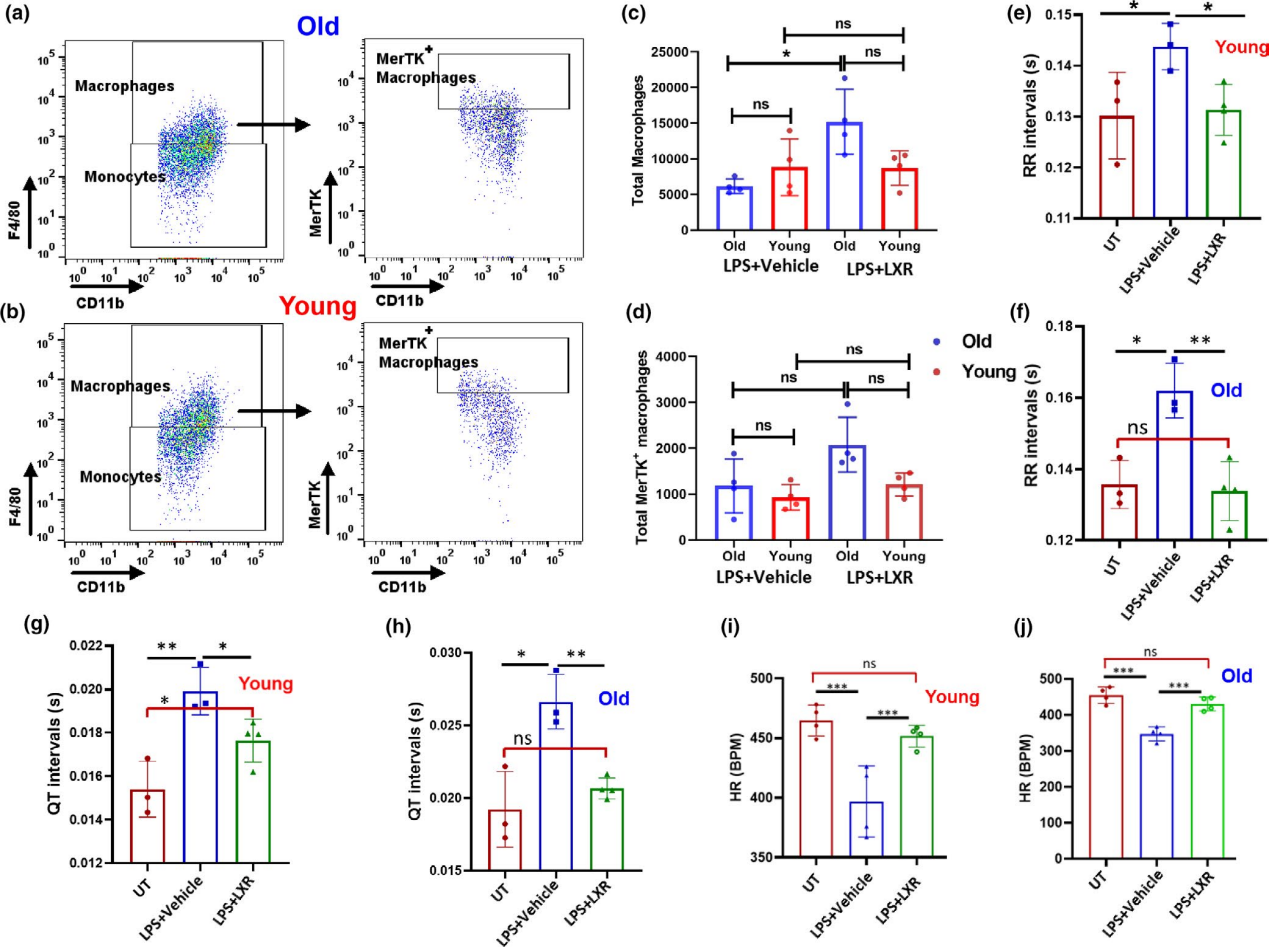
LXR agonist treatment enhances the number of MerTK^+^ CRMS and preserves the cardiac electrical function in old mice. Young and old female C57BL/6 mice were intraperitoneal injected either with T0901317 (50 mg/kg/day) or vehicle for 9 days in separate experiments. At day 10, mice were injected intraperitoneally with LPS (2.5 mg/kg) for 48 h and then euthanized and single‐cell suspensions were analyzed by flow cytometry: old (a) and young (b). Graphs shown in (c and d) are the total number of macrophages (CD45^+^CD11b^+^Ly6G^–^F4/80^+^) and MerTK^+^ macrophages in the heart tissue of vehicle and LXR agonist (T0901317) treated young and old mice followed by LPS injection, respectively. Surface electrocardiogram (ECG) recordings were obtained from control and LXR‐treated mice with and without LPS injection (2 mg/kg) of young and old mice at baseline and at 24 h post‐treatment by Powerlab 4/30 (AD Instruments). ECG traces were analyzed using LabChart 8 Pro (AD Instruments). Shown are representative graphs of RR intervals in young (e) and old (f) mice, QT intervals of young (g) and old (h) mice and heart rate of young (i) and old (j) mice. Data shown are representative of 5 mice (*N* = 5). A one‐way ANOVA followed by the Bonferroni test was used to analyze the data (*p < 0.05; **p < 0.005; and ***p < 0.0005)

Having determined that administration of LXR in young and old mice enhances the number of MerTK^+^ CRMs in the hearts, we repeated the experiment and tested whether LXR treatment protects the mice from LPS‐mediated cardiac electrical dysfunction. We administered LXR agonist or vehicle control to young and old mice for 12 days and subsequently delivered LPS via intraperitoneal route. The CRM phenotype, population, and heart function were examined as described above. Our flow cytometry data show that LXR treatment increased the number of MerTK^+^ CRMs in the hearts of young and old mice by 1.5‐fold compared with vehicle‐treated control mice (Figure 6a–d). These data suggests that the accumulation of MerTK^+^ CRMs in the heart plays an important role in maintaining the homeostasis function of heart by resolving the cardiac inflammation and tissue damage. Next, to confirm whether LXR treatment reverses the cardiac electrical dysfunction caused by LPS, we monitored the cardiac electrical activity (ECG) of mice pretreated with LXR agonist and infused with LPS. Our ECG data indicates that the LXR treatment reversed the LPS‐mediated increase of RR intervals, QT intervals, and heart rate in both young and old mice (Figure 6e–h). Most importantly we observed that LPS infusion caused arrhythmia (irregular QRS peaks with intermittent increase in RR intervals) and increased QT intervals (Figure S6) in both young and old mice, which was decreased by LXR agonist treatment. In conclusion, our data suggest that the LXR agonist is a potential target for cardio‐protection in old mice during cardiac infection or inflammation.

## 3 DISCUSSION

Since the elderly population is increasing, there is a greater need to understand age‐related changes and stressors (Franceschi et al., 2000). Although acute inflammation is important and helpful in protecting the body from infection, constant tissue damage, repair, and adaptation of tissues can lead to chronic, low‐grade inflammation. Such inflammation can be harmful by offsetting the normal functioning of tissues, ultimately leading to a decline in immune system function of elderly people (Calder et al., 2017). The elderly also commonly suffer from cardiovascular diseases, which are linked to dysregulated immune function (Bruunsgaard & Pedersen, 2003; Pawelec et al., 2014; Singh & Newman, 2011). Recently, the cardiac immune system has emerged as a critical player in maintaining steady‐state heart function, but the cardiac immune system is dysregulated by aging (Cieslik et al., 2011; Loffredo et al., 2014; Ramos et al., 2017). Myocardial aging is also associated with increased interstitial fibrosis, hypertrophy, inflammation, stiffness, and mild contractile dysfunction (Loffredo et al., 2014). Increased infiltration and differentiation of CD45^+^ cells of myeloid origin is the major driver for aging‐mediated cardiac dysfunction (Cieslik et al., 2011). Furthermore, we showed in prior studies that *M*. *avium*‐infected old mice had significant dysrhythmia, cardiac hypertrophy, increased recruitment of CD45^+^ leukocytes, cardiac fibrosis, and increased expression of inflammatory genes in heart tissue (Headley et al., 2019). To develop therapeutic strategies that ameliorate the inflammation that leads to cardiac dysfunction, it is crucial to understand the pathogenesis and mechanism of myocarditis induced by systemic inflammation.

In this study, we determined the effects of aging on the phenotype and function of cardiac immune cells and began to define the mechanisms by which aging causes cardiac inflammation in either the healthy condition or after low‐dose LPS treatment. We used a refined imaging approach to obtain accurate cell numbers and tissue distribution patterns for cardiac CD45^+^ cells in the steady‐state condition in old and young mice. We expanded the findings of Campos Ramos et al. (2017), which showed that age‐related myocardial impairment occurs in parallel with shifts in the composition of tissue‐resident leukocytes and with an accumulation of activated CD4^+^ T cells in the heart and draining lymph nodes. In line with those findings (Ramos et al., 2017), our morphological and functional analyses of cardiac immune cells of old and young mice revealed that the total number of cardiac leukocytes, including CD45^+^ cells, CD11b^+^ cells, neutrophils, and monocytes, was significantly higher in the hearts of old mice in a steady‐state condition compared with those of young mice. The study by Campos Ramos et al used echocardiography to investigate the relationship between the shifts in composition of leukocyte populations and the alterations in myocardial function in the aged heart (Ramos et al., 2017). They showed that there is a decrease in fractional shortening in aged mice compared with young mice (Ramos et al., 2017). Since an earlier report indicated that leukocyte infiltration into the heart alters the steady‐state heart function in aged mice, we examined whether systemic inflammation caused by LPS changes the cardiac immune cell phenotype and function. Our results showed a significant decrease in the infiltration of leukocytes, including CD45^+^ cells, CD11b^+^ cells, monocytes, and neutrophils into the hearts of LPS‐infused old mice. These observations support our premise that, although low‐grade cardiac inflammation in aged mice increases myeloid cells in the heart in a steady‐state condition, the increase in numbers does not have any effect on heart functionality in the steady‐state. However, the cardiac inflammation with aging makes the mice more susceptible to cardiac dysrhythmia upon LPS infusion, even though the LPS‐mediated systemic inflammation does not further increase myeloid cell numbers.

There is convincing evidence that aging is associated with increased production of myeloid cells, including MDSCs, in order to suppress inflammaging, which results in inimmunosenescence (Salminen et al., 2019). However, the infiltration of these specific cell types and their subsets (mMDSCs and gMDSCs) into the heart tissue of aged mice and their involvement in cardiac dysfunction has not been studied. Thus, in the current study, we compared total MDSC populations and their subsets in the hearts of old and young mice at steady‐state condition. In contrast to previous findings of higher frequencies of MDSCs in circulation (Salminen et al., 2019), we found that total MDSCs and more specifically, gMDSCs, were lower in the heart tissue of aged mice. While mMDSCs numbers did not differ between young and old mice, mMDSCs from old mice compared with mMDSCs from young mice were less able to suppress T‐cell proliferation ex vivo (Figure 2). In contrast, gMDSCs from young and old mice were equal in suppressing T‐cell proliferation. Thus, our data indicate that mMDSCs are deficient in suppressing T‐cell activation. This impairment becomes more critical in the case of HF or infection, which can lead to cardiac dysfunction. However, the literature concerning the role of MDSCs during HF, myocarditis, and infection is controversial (Cuervo et al., 2011; Fresno & Girones, 2018; Zhou et al., 2018). Zhou et al. reported that the number of MDSCs was significantly elevated during HF and correlated with increased levels of proinflammatory cytokines, given their compensatory role in resolving inflammation (Zhou et al., 2018). Furthermore, depletion of MDSCs aggravated cardiac hypertrophy, cardiac dysfunction, and inflammation, and increased T‐lymphocyte accumulation in the heart (Zhou et al., 2018). However, the adoptive transfer of MDSCs mitigated the overloaded pressure of cardiac hypertrophy and dysfunction (Zhou et al., 2018). Collectively, these findings suggest that MDSCs play a protective role during HF. In the case of infection, after pathogen recognition the myeloid cells generate an inflammatory response to clear the pathogen and develop adaptive immunity; then, MDSCs help repair tissue damage by suppressing inflammation. The expansion and/or activation of MDSC subsets that have regulatory activities play a critical role in these processes (Dorhoi et al., 2019). In a study using a mouse model of *Trypanosoma cruzi* infection, infiltrated Ly6G^+^ MDSCs (gMDSCs) into heart tissue triggered the recruitment of Ly6G^−^ MDSCs (mMDSCs) to the heart through the expression of S100A8/9 proteins and suppressed T‐cell proliferation by inducing arginase I and inducible nitric oxide synthase (Cuervo et al., 2011). In our model of aging, mMDSCs in the hearts of old mice are deficient in ability in inhibiting T‐cell proliferation. Thus, the inefficiency of mMDSCs function in old mice might contribute to the detrimental impact of chronic inflammation in the hearts of old mice.

Since the CRM phenotype and function are essential for the maintenance and homeostasis of myocardium under a steady‐state condition and during tissue‐repair processes (Epelman et al., 2014; Ma et al., 2018), we explored the phenotype of CRMs and its function in young and old mice in the context of infection. Previously, Campos Ramos et al. reported a clear decline in absolute numbers of macrophages in the heart due to aging (Ramos et al., 2017); moreover, we found that the numbers of total CRMs, regardless of their specific subset, did not decline with age in the steady‐state heart. However, the CRM population was significantly reduced in both young and old mice after LPS treatment. Furthermore, in‐depth analysis of CRM population in old and young mice, we found that MerTK^+^, MCH II high and low populations were significantly reduced in both old and young mice upon LPS infusion, which correlated with cardiac dysfunction (Figures 3 and 4). These observations raises questions as to whether macrophages are functionally able to resolve the excessive inflammation in the aged heart in steady state or after LPS infusion and by what mechanism they help regulate heart homeostasis. In homeostasis, it was suggested that embryonic precursor‐derived macrophage subsets are more practical for internalizing debris and for phagocytosis of apoptotic cardiomyocytes (Epelman et al., 2014; Lafuse et al., 2020). Considering that mechanism, Wan et al. reported that MerTK, a phagocytosis receptor, was upregulated on tissue macrophages during myocardial infarction (Wan et al., 2013). Conversely, a lack of MerTK expression can lead to accumulation of apoptotic cardiomyocytes, increased neutrophil persistence, and reduced levels of the anti‐inflammatory cytokine IL‐10 in the myocardium (Gautier et al., 2012). Here, we found that the number of CRMs expressing MerTK is reduced by LPS infusion in old and young mice. indicates that the loss of CRMs upon LPS infusion may play a critical role in causing more severe cardiac electrical dysfunction in old mice than young mice. Similar findings were reported by DeBerge et al. indicating that higher levels of MerTK on resident cardiac MHC‐II^low^ CCR2^−^ macrophages at steady state (DeBerge et al., 2017). They also reported that MerTK is cleaved from the surface of macrophages during ischemia and reperfusion (I/R) and that MerTK deficiency in macrophages attenuated phagocytosis, which resulted in a significant increase in neutrophils and Ly6C^high^ monocytes after I/R (DeBerge et al., 2017). Based on those findings, we hypothesize that the infiltration of monocytes into the heart of aged mice in a steady‐state condition might result in MerTK cleavage, which would reduce efferocytosis by CRMs and increase accumulation of neutrophils and inflammatory immune cells in the hearts of old mice. Thus, cardiac inflammation with aging could render the heart vulnerable to the effects of infection and unable to return to homeostasis, resulting in cardiac dysfunction during LPS infusion.

Given the beneficial effect of MerTK on the potential enhancement of cardiac repair during infection, we consider MerTK to be a new, promising target to improve heart tissue repair and dampen cardiac dysfunction after infections in the elderly. Zizzo et al. showed that, for the nuclear receptor superfamily members PPAR‐γ and LXR, inhibition of PPAR‐γ and activation of LXRs resulted in MerTK upregulation in human macrophages (Zizzo & Cohen, 2015). In another study, it was demonstrated that LXR signaling is not only critical for clearing apoptotic cells but is also pivotal for maintaining immune tolerance (Gonzalez et al., 2009). In our current in vitro study, we found that LXR activation but not PPAR‐γ inhibition resulted in increased MerTK expression in mouse BMDMs. Therefore, we examined the in vivo effects of LXR activation, in young and old mice, on expansion of the different CRM subsets as well as MerTK surface expression. We found that in vivo activation did not alter CRM subsets but substantially increased MerTK expression in MHC‐II^high/low^ CRMs (Figure 5). Since reduced phagocytosis and engulfment of apoptotic bodies occurs in macrophages from elderly humans and aged rodents in vitro and in vivo (Aprahamian et al., 2008; Izgut‐Uysal et al., 2004; Plowden et al., 2004), our studies suggest that LXR activation can be used to increase MerTK expression. In support of this notion, our data show that LXR pretreatment reversed the LPS‐mediated cardiac dysfunction in old and young mice, which correlates with an increased presence of MerTK‐expressing CRMs in the hearts of old and young mice (Figure 6). Thus, this approach to increase MerTK in CRMs and its function of clearance of apoptotic bodies from cardiac tissue might be a novel treatment for elderly people with chronic inflammatory diseases. The idea of using pharmacological LXR activation to prevent the progression of a wide range of inflammatory disorders has been well documented in preclinical animal models (Gonzalez et al., 2009; Fessler, 2018; Han et al., 2018). Additional research is needed so that this novel drug therapy could be used as a safe and effective treatment to prevent cardiac dysfunction due to infectious diseases, such as infection or chronic inflammation in the elderly.

In summary, our studies show that the hearts of old mice in the steady state have an inflammatory environment, with increased numbers of monocytes and neutrophils that makes them vulnerable to injuries caused by secondary factors, such as infections. We found that mMDSCs in old mice lack the ability to regulate immune T cells, which may also contribute to an inflammatory environment. However, the numbers of CRMs did not change with aging, indicating that inflammaging does not alter the resident cardiac macrophage population. After infusion of a low dose of LPS, the numbers of CRMs decreased in both young and old mice. This decline affected MerTK^+^ CRMs, as well as MHC‐II^high^ and MHC‐II^low^ CRMs, which was accompanied by cardiac dysfunction that was more pronounced in old mice. While it has been established that cardiac dysfunction occurs during sepsis, our studies show that even a sub‐lethal dose of LPS alters the cardiac macrophage populations and causes cardiac dysfunction. Our studies also show that treating mice with an LXR activator increases MerKT expression on cardiac macrophages and decreases cardiac dysfunction induced by LPS. Thus, to prevent the cardiac electrical dysfunction accompanied with aging that occurs during infections or endotoxin shock, we propose the use of an LXR activator, which leads to the increased expression of MerTK in the heart tissue and helps to maintain homeostasis and protect the heart.

## 4 MATERIALS AND METHODS

### 4.1 Mice

C57BL/6 mice were purchased from Charles River Laboratories (Wilmington, MA) at age 6–8 weeks (young) or at 18 months (Pawelec et al), through a contract with the National Institute of Aging. Mice were housed in microisolator cages of 3–5 mice/cage and acclimated to the facility for 1 week prior to use. Mice were maintained on a 12‐h light/dark schedule, and food and water were available to them *ad libitum*. All procedures were approved by The Ohio State University Institutional Laboratory Animal Care and Use Committee.

### 4.2 Heart tissue digestion and single‐cell preparation

Mice were euthanized by CO_2_ inhalation. Heart single cells were prepared according to the protocol of Nahrendorf et al. (2007) with modification as described below.

Hearts were perfused with 20 ml of cold PBS, weighed, minced, and then digested using a Multi Tissue Dissociation Kit 2 and gentleMACS™ Dissociator (MiltenyiBiotec) according to the manufacturer's instructions. Briefly, perfused hearts were cut into small pieces (1–2 mm^2^), placed in gentleMACS C‐tubes containing 2.5 ml of enzyme mix, incubated at 37℃ for 15 min without agitation, and then dissociated using the Multi‐G program of a gentleMACS Dissociator (Meeson et al., 2013). These steps were then repeated twice. After the program was terminated, the C‐tube was detached from the gentleMACS Dissociator, 7.5 ml of cell culture medium with 20% FBS was added, and the cells passed through a MACS Smart Strainer (70 µm) and centrifuged to collect single cells. Red blood cells were removed by resuspending cells in red blood cell lysis buffer and washed with 3ml of cell culture medium with 20% FBS. The single‐cell suspensions were resuspended with appropriate buffer or medium, counted, and used for further applications. Detailed gating strategies, antibodies used and data analysis can be found in the Supplemental Information section.

### 4.3 Statistical analysis and Study approval

The data are presented as mean ± standard error of mean. A Student's two‐tailed *t* test was used to compare two groups. A one‐way ANOVA followed by the Bonferroni test was used to compare three or more groups. All statistical analyses were performed using Graph Pad Prism software. *p* < 0.05 was considered statistically significant. Animal use in this study was approved by the Institutional Biosafety Committee and IACUC of the Ohio State University and adhered to NIH guidelines for the use of experimental animals.

## CONFLICT OF INTEREST

The authors have declared that no conflict of interests exists.

## AUTHOR CONTRIBUTIONS

NS and MVSR conceived the ideas and designed the experiments. NS, QW, NK, LPG, WPL, and MVSR performed most of the experiments, acquired, and analyzed the data. NS, WPL, and MVSR wrote the manuscript. LPG, WPL, and MVSR edited the manuscript.

## Supporting information

Supplementary Material

## Data Availability

The authors do not include any large data sets in this manuscript.

## References

[acel13438-bib-0001] A‐Gonzalez, N., Bensinger, S. J., Hong, C., Beceiro, S., Bradley, M. N., Zelcer, N., Deniz, J., Ramirez, C., Díaz, M., Gallardo, G., Ruiz de Galarreta, C., Salazar, J., Lopez, F., Edwards, P., Parks, J., Andujar, M., Tontonoz, P., & Castrillo, A. (2009). Apoptotic cells promote their own clearance and immune tolerance through activation of the nuclear receptor LXR. Immunity, 31(2), 245–258. 10.1016/j.immuni.2009.06.018 19646905 PMC2791787

[acel13438-bib-0002] Aprahamian, T., Takemura, Y., Goukassian, D., & Walsh, K. (2008). Ageing is associated with diminished apoptotic cell clearance in vivo. Clinical and Experimental Immunology, 152(3), 448–455. 10.1111/j.1365-2249.2008.03658.x 18422728 PMC2453212

[acel13438-bib-0003] Bruunsgaard, H., & Pedersen, B. K. (2003). Age‐related inflammatory cytokines and disease. Immunology and Allergy Clinics of North America, 23(1), 15–39. 10.1016/S0889-8561(02)00056-5 12645876

[acel13438-bib-0004] Calder, P. C., Bosco, N., Bourdet‐Sicard, R., Capuron, L., Delzenne, N., Doré, J., Franceschi, C., Lehtinen, M. J., Recker, T., Salvioli, S., & Visioli, F. (2017). Health relevance of the modification of low grade inflammation in ageing (inflammageing) and the role of nutrition. Ageing Research Reviews, 40, 95–119. 10.1016/j.arr.2017.09.001 28899766

[acel13438-bib-0005] Cevenini, E., Monti, D., & Franceschi, C. (2013). Inflamm‐ageing. Current Opinion in Clinical Nutrition and Metabolic Care, 16(1), 14–20. 10.1097/MCO.0b013e32835ada13 23132168

[acel13438-bib-0006] Cieslik, K. A., Taffet, G. E., Carlson, S., Hermosillo, J., Trial, J., & Entman, M. L. (2011). Immune‐inflammatory dysregulation modulates the incidence of progressive fibrosis and diastolic stiffness in the aging heart. Journal of Molecular and Cellular Cardiology, 50(1), 248–256. 10.1016/j.yjmcc.2010.10.019 20974150 PMC3019252

[acel13438-bib-0007] Costantino, S., Paneni, F., & Cosentino, F. (2016). Ageing, metabolism and cardiovascular disease. Journal of Physiology, 594(8), 2061–2073. 10.1113/JP270538 PMC4933114 26391109

[acel13438-bib-0008] Cuervo, H., Guerrero, N. A., Carbajosa, S., Beschin, A., De Baetselier, P., Girones, N., & Fresno, M. (2011). Myeloid‐derived suppressor cells infiltrate the heart in acute trypanosoma cruzi infection. The Journal of Immunology, 187(5), 2656–2665. 10.4049/jimmunol.1002928 21804013

[acel13438-bib-0009] de Almeida, A. J. P. O., de Almeida Rezende, M. S., Dantas, S. H., de Lima Silva, S., de Oliveira, J. C. P. L., de Lourdes Assunção Araújo de Azevedo, F., Alves, R. M. F. R., de Menezes, G. M. S., dos Santos, P. F., Gonçalves, T. A. F., Schini‐Kerth, V. B., & de Medeiros, I. A. (2020). Unveiling the role of inflammation and oxidative stress on age‐related cardiovascular diseases. Oxidative Medicine and Cellular Longevity, 2020, 1954398. 10.1155/2020/1954398 32454933 PMC7232723

[acel13438-bib-0010] DeBerge, M., Yeap, X. Y., Dehn, S., Zhang, S., Grigoryeva, L., Misener, S., Procissi, D., Zhou, X., Lee, D. C., Muller, W. A., Luo, X., Rothlin, C., Tabas, I., & Thorp, E. B. (2017). MerTK cleavage on resident cardiac macrophages compromises repair after myocardial ischemia reperfusion injury. Circulation Research, 121(8), 930–940. 10.1161/CIRCRESAHA.117.311327 28851810 PMC5623080

[acel13438-bib-0011] Demissei, B. G., Cleland, J. G., O'Connor, C. M., Metra, M., Ponikowski, P., Teerlink, J. R., Davison, B., Givertz, M. M., Bloomfield, D. M., Dittrich, H., van Veldhuisen, D. J., Hillege, H. L., Voors, A. A., & Cotter, G. (2016). Procalcitonin‐based indication of bacterial infection identifies high risk acute heart failure patients. International Journal of Cardiology, 204, 164–171. 10.1016/j.ijcard.2015.11.141 26666342

[acel13438-bib-0012] Deutschman, C. S., & Tracey, K. J. (2014). Sepsis: Current dogma and new perspectives. Immunity, 40(4), 463–475. 10.1016/j.immuni.2014.04.001 24745331

[acel13438-bib-0013] Diez‐Villanueva, P., & Alfonso, F. (2016). Heart failure in the elderly. Journal of Geriatric Cardiology, 13(2), 115–117. 10.11909/j.issn.1671-5411.2016.02.009 27168735 PMC4854948

[acel13438-bib-0014] Dorhoi, A., Glaría, E., Garcia‐Tellez, T., Nieuwenhuizen, N. E., Zelinskyy, G., Favier, B., Singh, A., Ehrchen, J., Gujer, C., Münz, C., Saraiva, M., Sohrabi, Y., Sousa, A. E., Delputte, P., Müller‐Trutwin, M., & Valledor, A. F. (2019). MDSCs in infectious diseases: regulation, roles, and readjustment. Cancer Immunology, Immunotherapy, 68(4), 673–685. 10.1007/s00262-018-2277-y 30569204 PMC11028159

[acel13438-bib-0015] Drosatos, K., Khan, R. S., Trent, C. M., Jiang, H., Son, N. H., Blaner, W. S., & Goldberg, I. J. (2013). Peroxisome proliferator‐activated receptor‐gamma activation prevents sepsisrelated cardiac dysfunction and mortality in mice. Circulation: Heart Failure, 6(3), 550–562. 10.1161/CIRCHEARTFAILURE.112.000177 23572494 PMC3690188

[acel13438-bib-0016] Epelman, S., Lavine, K. J., Beaudin, A. E., Sojka, D. K., Carrero, J. A., Calderon, B., Brija, T., Gautier, E. L., Ivanov, S., Satpathy, A. T., Schilling, J. D., Schwendener, R., Sergin, I., Razani, B., Forsberg, E. C., Yokoyama, W. M., Unanue, E. R., Colonna, M., Randolph, G. J., & Mann, D. L. (2014). Embryonic and adult‐derived resident cardiac macrophages are maintained through distinct mechanisms at steady state and during inflammation. Immunity, 40(1), 1–104. 10.1016/j.immuni.2013.11.019 24439267 PMC3923301

[acel13438-bib-0017] Epelman, S., Liu, P. P., & Mann, D. L. (2015). Role of innate and adaptive immune mechanisms in cardiac injury and repair. Nature Reviews Immunology, 15(2), 117–129. 10.1038/nri3800 PMC4669103 25614321

[acel13438-bib-0018] Fallach, R., Shainberg, A., Avlas, O., Fainblut, M., Chepurko, Y., Porat, E., & Hochhauser, E. (2010). Cardiomyocyte toll‐like receptor 4 is involved in heart dysfunction following septic shock or myocardial ischemia. Journal of Molecular and Cellular Cardiology, 48(6), 1236–1244. 10.1016/j.yjmcc.2010.02.020 20211628

[acel13438-bib-0019] Fessler, M. B. (2018). The challenges and promise of targeting the liver X receptors for treatment of inflammatory disease. Pharmacology & Therapeutics, 181, 1–12. 10.1016/j.pharmthera.2017.07.010 28720427 PMC5743771

[acel13438-bib-0020] Franceschi, C., Bonafe, M., Valensin, S., Olivieri, F., De Luca, M., Ottaviani, E., & De Benedictis, G. (2000). Inflamm‐aging: An evolutionary perspective on immunosenescence. Annals of the New York Academy of Sciences, 908, 244–254. 10.1111/j.1749-6632.2000.tb06651.x 10911963

[acel13438-bib-0021] Fresno, M., & Girones, N. (2018). Regulatory lymphoid and myeloid cells determine the cardiac immunopathogenesis of Trypanosoma cruzi infection. Frontiers in Microbiology, 9, 351. 10.3389/fmicb.2018.00351 29545782 PMC5838393

[acel13438-bib-0022] Gao, M., & Liu, D. (2013). The liver X receptor agonist T0901317 protects mice from high fat diet‐induced obesity and insulin resistance. American Association of Pharmaceutical Scientists Journal, 15(1), 258–266. 10.1208/s12248-012-9429-3 23180161 PMC3535091

[acel13438-bib-0023] Gautier, E. L., Shay, T., Miller, J., Greter, M., Jakubzick, C., Ivanov, S., Helft, J., Chow, A., Elpek, K. G., Gordonov, S., Mazloom, A. R., Ma'ayan, A., Chua, W.‐J., Hansen, T. H., Turley, S. J., Merad, M., & Randolph, G. J. (2012). Gene‐expression profiles and transcriptional regulatory pathways that underlie the identity and diversity of mouse tissue macrophages. Nature Immunology, 13(11), 1118–1128. 10.1038/ni.2419 23023392 PMC3558276

[acel13438-bib-0024] Greifenberg, V., Ribechini, E., Rossner, S., & Lutz, M. B. (2009). Myeloid‐derived suppressor cell activation by combined LPS and IFN‐gamma treatment impairs DC development. European Journal of Immunology, 39(10), 2865–2876. 10.1002/eji.200939486 19637228

[acel13438-bib-0025] Han, S., Zhuang, H., Shumyak, S., Wu, J., Xie, C., Li, H., Yang, L.‐J., & Reeves, W. H. (2018). Liver X receptor agonist therapy prevents diffuse alveolar hemorrhage in murine lupus by repolarizing macrophages. Frontiers in Immunology, 9, 135. 10.3389/fimmu.2018.00135 29456535 PMC5801423

[acel13438-bib-0026] Headley, C. A., Gerberick, A., Mehta, S., Wu, Q., Yu, L., Fadda, P., & Rajaram, M. V. S. (2019). Nontuberculous mycobacterium M. avium infection predisposes aged mice to cardiac abnormalities and inflammation. Aging Cell, 18(3), e12926. 10.1111/acel.12926 30834643 PMC6516181

[acel13438-bib-0027] Hüsecken, Y., Muche, S., Kustermann, M., Klingspor, M., Palmer, A., Braumüller, S., Huber‐Lang, M., Debatin, K.‐M., & Strauss, G. (2017). MDSCs are induced after experimental blunt chest trauma and subsequently alter antigen‐specific T cell responses. Scientific Reports, 7(1), 12808. 10.1038/s41598-017-13019-6 28993671 PMC5634472

[acel13438-bib-0028] Izgut‐Uysal, V. N., Ozkaya, Y. G., Ozdemir, S., Yargicoglu, P., & Agar, A. (2004). Effect of L‐Arginine on age‐related changes in macrophage phagocytic activity. Immunological Investigations, 33(3), 287–293. 10.1081/imm-120037276 15495788

[acel13438-bib-0029] Kovacic, J. C., Moreno, P., Nabel, E. G., Hachinski, V., & Fuster, V. (2011). Cellular senescence, vascular disease, and aging: part 2 of a 2‐part review: Clinical vascular disease in the elderly. Circulation, 123(17), 1900–1910. 10.1161/CIRCULATIONAHA.110.009118 21537006

[acel13438-bib-0030] Lafuse, W. P., Wozniak, D. J., & Rajaram, M. V. S. (2020). Role of cardiac macrophages on cardiac inflammation, fibrosis and tissue repair. Cells, 10(1), 51. 10.3390/cells10010051 PMC7824389 33396359

[acel13438-bib-0031] Lemke, G., & Rothlin, C. V. (2008). Immunobiology of the TAM receptors. Nature Reviews Immunology, 8(5), 327–336. 10.1038/nri2303 PMC2856445 18421305

[acel13438-bib-0032] Loffredo, F. S., Nikolova, A. P., Pancoast, J. R., & Lee, R. T. (2014). Heart failure with preserved ejection fraction: Molecular pathways of the aging myocardium. Circulation Research, 115(1), 97–107. 10.1161/CIRCRESAHA.115.302929 24951760 PMC4094348

[acel13438-bib-0033] Ma, Y., Mouton, A. J., & Lindsey, M. L. (2018). Cardiac macrophage biology in the steady‐state heart, the aging heart, and following myocardial infarction. Translational Research, 191, 15–28. 10.1016/j.trsl.2017.10.001 29106912 PMC5846093

[acel13438-bib-0034] Makara, M. A., Hoang, K. V., Ganesan, L. P., Crouser, E. D., Khan, M., Gunn, J. S., Turner, J., Schlesinger, L. S., Mohler, P. J., & Rajaram, M. V. S. (2016). Cardiac electrical and structural changes during bacterial infection: An instructive model to study cardiac dysfunction in sepsis. Journal of the American Heart Association, 5(9). 10.1161/JAHA.116.003820 PMC5079037 27620887

[acel13438-bib-0035] Martin, G. S., Mannino, D. M., & Moss, M. (2006). The effect of age on the development and outcome of adult sepsis. Critical Care Medicine, 34(1), 15–21. 10.1097/01.ccm.0000194535.82812.ba 16374151

[acel13438-bib-0036] Mayr, F. B., Yende, S., Linde‐Zwirble, W. T., Peck‐Palmer, O. M., Barnato, A. E., Weissfeld, L. A., & Angus, D. C. (2010). Infection rate and acute organ dysfunction risk as explanations for racial differences in severe sepsis. JAMA, 303(24), 2495–2503. 10.1001/jama.2010.851 20571016 PMC3910506

[acel13438-bib-0037] Meeson, A., Fuller, A., Breault, D. T., Owens, W. A., & Richardson, G. D. (2013). Optimised protocols for the identification of the murine cardiac side population. Stem Cell Reviews and Reports, 9(5), 731–739. 10.1007/s12015-013-9440-9 23619929 PMC4629417

[acel13438-bib-0038] Meschiari, C. A., Ero, O. K., Pan, H., Finkel, T., & Lindsey, M. L. (2017). The impact of aging on cardiac extracellular matrix. Geroscience, 39(1), 7–18. 10.1007/s11357-017-9959-9 28299638 PMC5352584

[acel13438-bib-0039] Nahrendorf, M., Swirski, F. K., Aikawa, E., Stangenberg, L., Wurdinger, T., Figueiredo, J.‐L., Libby, P., Weissleder, R., & Pittet, M. J. (2007). The healing myocardium sequentially mobilizes two monocyte subsets with divergent and complementary functions. Journal of Experimental Medicine, 204(12), 3037–3047. 10.1084/jem.20070885 PMC2118517 18025128

[acel13438-bib-0040] Oh, Y. J., Pau, V. C., Steppan, J., Sikka, G., Bead, V. R., Nyhan, D., Levine, B. D., Berkowitz, D. E., & Santhanam, L. (2017). Role of tissue transglutaminase in age‐associated ventricular stiffness. Amino Acids, 49(3), 695–704. 10.1007/s00726-016-2295-z 27438265

[acel13438-bib-0041] Panth, N., Paudel, K. R., & Parajuli, K. (2016). Reactive oxygen species: A key hallmark of cardiovascular disease. Advances in Medicine, 2016, 1–12. 10.1155/2016/9152732 PMC5059509 27774507

[acel13438-bib-0042] Papaconstantinou, J. (2019). The role of signaling pathways of inflammation and oxidative stress in development of senescence and aging phenotypes in cardiovascular disease. Cells, 8(11). 10.3390/cells8111383 PMC6912541 31689891

[acel13438-bib-0043] Parker, M. M., Shelhamer, J. H., Bacharach, S. L., Green, M. V., Natanson, C., Frederick, T. M., & Parrillo, J. E. (1984). Profound but reversible myocardial depression in patients with septic shock. Annals of Internal Medicine, 100(4), 483–490. 10.7326/0003-4819-100-4-483 6703540

[acel13438-bib-0044] Pawelec, G., Goldeck, D., & Derhovanessian, E. (2014). Inflammation, ageing and chronic disease. Current Opinion in Immunology, 29, 23–28. 10.1016/j.coi.2014.03.007 24762450

[acel13438-bib-0045] Penberthy, K. K., & Ravichandran, K. S. (2016). Apoptotic cell recognition receptors and scavenger receptors. Immunological Reviews, 269(1), 44–59. 10.1111/imr.12376 26683144 PMC4685734

[acel13438-bib-0046] Plowden, J., Renshaw‐Hoelscher, M., Engleman, C., Katz, J., & Sambhara, S. (2004). Innate immunity in aging: Impact on macrophage function. Aging Cell, 3(4), 161–167. 10.1111/j.1474-9728.2004.00102.x 15268749

[acel13438-bib-0047] Ramos, G. C., van den Berg, A., Nunes‐Silva, V., Weirather, J., Peters, L., Burkard, M., Friedrich, M., Pinnecker, J., Abeßer, M., Heinze, K. G., Schuh, K., Beyersdorf, N., Kerkau, T., Demengeot, J., Frantz, S., & Hofmann, U. (2017). Myocardial aging as a T‐cell‐mediated phenomenon. Proceedings of the National Academy of Sciences United States of America, 114(12), E2420–E2429. 10.1073/pnas.1621047114 PMC5373357 28255084

[acel13438-bib-0048] Romero‐Bermejo, F. J., Ruiz‐Bailen, M., Gil‐Cebrian, J., & Huertos‐Ranchal, M. J. (2011). Sepsis‐induced cardiomyopathy. Current Cardiology Reviews, 7(3), 163–183. 10.2174/157340311798220494 22758615 PMC3263481

[acel13438-bib-0049] Ryzhov, S., Novitskiy, S. V., Goldstein, A. E., Biktasova, A., Blackburn, M. R., Biaggioni, I., & Feoktistov, I. (2011). Adenosinergic regulation of the expansion and immunosuppressive activity of CD11b+Gr1+ cells. The Journal of Immunology, 187(11), 6120–6129. 10.4049/jimmunol.1101225 22039302 PMC3221925

[acel13438-bib-0050] Salminen, A., Kaarniranta, K., & Kauppinen, A. (2019). Immunosenescence: The potential role of myeloid‐derived suppressor cells (MDSC) in age‐related immune deficiency. Cellular and Molecular Life Sciences, 76(10), 1901–1918. 10.1007/s00018-019-03048-x 30788516 PMC6478639

[acel13438-bib-0051] Sies, H. (2015). Oxidative stress: A concept in redox biology and medicine. Redox Biology, 4, 180–183. 10.1016/j.redox.2015.01.002 25588755 PMC4309861

[acel13438-bib-0052] Singh, T., & Newman, A. B. (2011). Inflammatory markers in population studies of aging. Ageing Research Reviews, 10(3), 319–329. 10.1016/j.arr.2010.11.002 21145432 PMC3098911

[acel13438-bib-0053] Smit, J., Adelborg, K., Thomsen, R. W., Sogaard, M., & Schonheyder, H. C. (2016). Chronic heart failure and mortality in patients with community‐acquired Staphylococcus aureus bacteremia: A population‐based cohort study. BMC Infectious Diseases, 16, 227. 10.1186/s12879-016-1570-7 27225712 PMC4880885

[acel13438-bib-0054] United Nations, Department of Economic and Social Affairs, Population Division . (2017). World Population Ageing 2017 ‐ Highlights ST/ESA/SER.A/397.

[acel13438-bib-0055] van der Poll, T., van de Veerdonk, F. L., Scicluna, B. P., & Netea, M. G. (2017). The immunopathology of sepsis and potential therapeutic targets. Nature Reviews Immunology, 17(7), 407–420. 10.1038/nri.2017.36 28436424

[acel13438-bib-0056] Wan, E., Yeap, X. Y., Dehn, S., Terry, R., Novak, M., Zhang, S., & Thorp, E. B. (2013). Enhanced efferocytosis of apoptotic cardiomyocytes through myeloid‐epithelialreproductive tyrosine kinase links acute inflammation resolution to cardiac repair after infarction. Circulation Research, 113(8), 1004–1012. 10.1161/CIRCRESAHA.113.301198 23836795 PMC3840464

[acel13438-bib-0057] Wang, W., Zhang, X., Ge, N. A., Liu, J., Yuan, H., Zhang, P., Liu, W., & Wen, D. (2014). Procalcitonin testing for diagnosis and short‐term prognosis in bacterial infection complicated by congestive heart failure: A multicenter analysis of 4,698 cases. Critical Care, 18(1), R4. 10.1186/cc13181 24393388 PMC4056105

[acel13438-bib-0058] Wu, J., Xia, S., Kalionis, B., Wan, W., & Sun, T. (2014). The role of oxidative stress and inflammation in cardiovascular aging. BioMed Research International, 2014, 1–13. 10.1155/2014/615312 PMC4131065 25143940

[acel13438-bib-0059] Zhou, L., Miao, K., Yin, B., Li, H., Fan, J., Zhu, Y., Ba, H., Zhang, Z., Chen, F., Wang, J., Zhao, C., Li, Z., & Wang, D. W. (2018). Cardioprotective role of myeloid‐derived suppressor cells in heart failure. Circulation, 138(2), 181–197. 10.1161/CIRCULATIONAHA.117.030811 29437117

[acel13438-bib-0060] Zizzo, G., & Cohen, P. L. (2015). The PPAR‐gamma antagonist GW9662 elicits differentiation of M2c‐like cells and upregulation of the MerTK/Gas6 axis: A key role for PPAR‐gamma in human macrophage polarization. Journal of Inflammation (London), 12, 36. 10.1186/s12950-0150081-4 PMC4429687 25972766

