# Supplementary material for: Aging influences the cardiac macrophage phenotype and function during steady state and during inflammation
Source: Aging Cell. 2021 Aug 2;20(8):e13438. doi: 10.1111/acel.13438 (PMC8373275; doi:10.1111/acel.13438)
Supplement: Supplementary file 1 — Supplementary Material [file ACEL-20-e13438-s001.docx]

**Supplemental Information:**

*Lipopolysaccharide (LPS) instillation of mice*

Young (6-8 weeks) and old (18 months) female C57BL/6 mice were sedated with isoflurane, then injected intraperitoneally with sub-lethal dose of LPS (2.5 mg/Kg)*.* The infused young and old mice were euthanized on 48h for cardiac immune cell phenotyping. We euthanized the wild type young and old mice as a control. In a separate set of experiments, young female C57BL/6 mice were treated with the liver X receptor (LXR) agonist T0901317 (50mg/kg/day)(Cayman Chemicals) or vehicle (10% Cremaphore; EMD Millipore) in saline daily via intraperitoneal route for 9 days. The LXR agonist treated or untreated young and old mice were infused with LPS via intraperitoneal route (2.5 mg/kg) in saline.

*Flow cytometry analysis of cardiac single cells*

Heart single-cell suspensions were aliquoted into FACS tubes and centrifuged at 250x *g* for 10 min. Cell pellets were suspended in 100µl of FACS buffer (PBS buffer with 2% BSA and 0.10% sodium azide) and 2 µl of anti-mouse CD16/CD32 (Bio-Rad Laboratories) added to block Fc receptors. After 15 min of incubation at room temperature, cells were stained with Abs for 30 min in the dark at 4°C. Cells were washed twice and resuspended in FACS buffer and incubated with the following antibodies from BioLegend: PERCP/Cy5.5 anti-CD45.2 (clone104), BV421 anti-CD11b (clone M1/70), BV605 anti-Ly6G (clone1A8), PE anti-F4/80 (cloneM8), APC/Fire 750 anti-I-A/I-E (clone M5/114.15.2), FITC anti-MerTK (clone 2B10C42), APC anti-Ly-6C (cloneHK1.4), Brilliant Violet 510 anti-CD192 (CCR2) (cloneSA203G11), PE anti-Ly-6G/Ly-6C (Gr-1) (cloneRB6-8C5), Alexa Fluor 488 anti-Ly6G (clone1A8), PE anti-CD3ε (clone 145-2C11), Alexa Fluor 488 anti-CD8a (clone53-6.7), and Brilliant Violet 605 anti-CD4 (cloneRM4-4). Isotype controls were APC/Fire 750 Rat IgG2b, Brilliant Violet 510 Rat IgG2b, Brilliant Violet 605 Rat IgG2a, APC Rat IgG2c, and FITC Rat IgG2a. Stained cell suspensions were processed on a BD LSR Fortessa™ cell analyzer (BD Biosciences) and output analyzed using FlowJo software (Tree Star). Specific mean fluorescence intensity was calculated by subtracting the isotype MFI from the Ab-specific MFI. Populations were gated using fluorescence-minus-one controls. After gating on CD45^+^ cells, doublets were excluded, and live cells were analyzed using forward scatter/side scatter live-dead exclusion. To analyze myeloid cells, lymphocytes were first excluded using a lin^-^ cocktail of antibodies (Alexa Fluor 488 anti-CD45R/B220 [Clone RA3-6B2], anti-CD90.2 [Clone30-H12], anti-mouse NK1.1 [ClonePK136], and anti-TER119/Erythroid Cells[CloneTER-119]).The gating strategy of cardiac myeloid cells was as follows: total cardiac myeloid cells: CD45^+^CD11b^+^, neutrophils: CD45^+^CD11b^+^Ly6G^+^, infiltrating monocytes: CD45^+^CD11b^+^Ly6G^-^F4/80^-^Ly6C^high/low^, and cardiac macrophages: CD45^+^CD11b^+^Ly6G^-^F4/80^+^MHC-II^high/low^ CCR2^+^ or CCR2^-^and MerTK^+^. MDSCs were gated as follows: total MDSCs: CD45^+^CD11b^+^Gr-1^int/low^, mMDSCs: CD45^+^CD11b^+^Gr-1^low^Ly6C^+^Ly6G^-^, and granulocyte-derived MDSCs: CD45^+^CD11b^+^Gr-1^int^Ly6C^low^Ly6G^+^. Single-cell analysis software (FlowJo 10.2) was used to analyze cell cytometric data. To generate cluster plots, cells were pre-gated on CD45^+^CD11b^+^Ly6G^–^F4/80^+^, and populations derived from several animals of each group were subjected to simultaneous T-distributed stochastic neighbor embedding (t-SNE) analysis. The following parameters were used: iterations, 1000; perplexity, 30; and Eta, 410. To quantify the differences in MerTK expression on the histogram overlays, a geometric mean statistic for FITC-MerTK was added to each MHCII^high/low^ CCR2^+/-^ subset, giving a measurement of central tendency in the FITC-MerTK channel, which represents relative expression of MerTK per cell in each subset population.

*Proliferation assay*

Myeloid-derived suppressor cells (MDSCs) were separated from single-cell suspensions of heart tissue from young (6-8 weeks) and old (18 months) female C57BL/6 mice. Briefly, single-cell suspensions of murine heart tissue were prepared using the Multi Tissue Dissociation Kit and gentle-MACsDissociator (Miltenyi Biotec). MDSCs were isolated using the Myeloid-Derived Suppressor Cell Isolation Kit (Miltenyi Biotec). GR1^high^ Ly6G^+^ and GR1^dim^ Ly6G^-^ MDSCs were isolated according to the manufacturer’s instructions. CD4^+^ T cells were isolated from spleens of 3 young (6-8 weeks) female C57BL/6 mice using a Naïve CD4^+^ T Cell Isolation Kit (Miltenyi Biotec) according to the manufacturer’s instructions and stained with CFSE (10 µM, Invitrogen). A 96-well plate was prepared by adding 50 µl of Mouse T-Activator (CD3/CD28 Dynabeads™ (Gibco™) for 2 h at 37°C. Then, CD4^+^ T cells were co-cultured with GR1^high^ Ly6G^+^ or GR1^dim^ Ly6G^-^ MDSCs (ratio 2:1) for 72 h at 37°C.The cells were removed, CD4^+^ T cells were stained with CD4^+^ antibody, and proliferation was analyzed by dilution of CFSE using flow cytometry.

*Generation of bone marrow–derived macrophages*

Bone marrow precursor cells were isolated from femurs and tibias of C57BL/6 mice for differentiation into bone marrow–derived macrophages (BMDMs). Briefly, bone marrow cells were flushed from femurs and tibias with HBSS (100 U/ml penicillin, 100 μg/ml streptomycin, 4.2 mM sodium bicarbonate, 20 mM HEPES) at 4°C and centrifuged. Bone marrow cells were then suspended in BMDM culture medium (DMEM, 10% heat-inactivated FBS, 0.6% nonessential amino acids, 100 U/ml penicillin, 100 μg/ml streptomycin) supplemented with 50% L929 fibroblast-conditioned culture medium (L Cell Conditioned Medium). Cells were seeded in 15-cm-diameter tissue culture–treated dishes and incubated at 37°C and 5% CO_2_. On day 3, BMDM culture medium was added (∼10 ml/dish). The BMDMs were harvested on day 5. The differentiation of precursor cells into macrophages was routinely assessed by monitoring CD11b and F4/80 co-expression by flow cytometry.

*RNA isolation and qRT-PCR*

BMDM cells were cultured overnight in 12-well tissue culture plates at 0.6×10^6^ cells/well. Then, non-adherent cells were removed and BMDMs were treated with either DMSO, GW9662 (PPAR-γ antagonist, 10 μM), or T0901317 (LXR agonist, 1 μM) (Cayman Chemical, Ann Arbor, MI, USA) at 37°C for 24 h in the presence of 5% CO_2_. BMDMs were lysed using Trizol Reagent (Life Technologies), and their RNA was extracted with chloroform and precipitated with isopropanol. RNA pellets were washed once with 75% ethanol and reconstituted with 30µl RNase-free water. Samples (800 ng of RNA/sample) were digested with DNase (QIAGEN) prior to reverse transcription using the SuperScript™ III Reverse Transcriptase (Invitrogen). The resultant cDNA was used to determine the expression of MerTK by quantitative RT-PCR (qRT-PCR) using TaqMan Gene Expression Assays ([Applied Biosystems](http://www.thermofisher.com/us/en/home/brands/applied-biosystems.html)) and normalized to β-actin (*actb)* as a housekeeping gene. The mRNA levels in treated BMDMs were expressed relative to control untreated BMDMs.

*Cell lysis and western blotting*

After treatment, BMDM monolayers were washed once in PBS and lysed in TN-1 lysis buffer at 4°C for 10 min and centrifuged at 10,000 × *g* for 10 min at 4°C to remove cell debris (1). Protein concentrations of the cleared lysates were measured by the BCA Protein Assay Kit (Pierce). Protein-matched total cell lysates were separated by SDS-PAGE and analyzed by western blot using the following Abs: rabbit anti-MerTK antibody (ab196880) from Abcam and β-actin antibody (Santa Cruz). The protein band intensities were measured using the online ImageJ software provided by the National Institutes of Health. Background intensity was subtracted from each sample and then normalized to the β-actin as loading control.

*Electrocardiography Recordings*

At day 10 post-LXR treatment with and without LPS infusion subsurface Electrocardiogram (ECG) were recorded. Young and old treated mice and aged matched control (sham) mice were anesthetized using 2% isoflurane in oxygen (flow rate 1.0 L/min), which was subsequently lowered to 1% (1.0 L/min) for the duration of the ECG recordings. Mice were placed in the prone position and kept on a heated pad to maintain body temperature. The subcutaneous electrodes for ECG were placed in the lead II configuration and ECGs were recorded for 10 minutes on a Powerlab 4/30 (AD Instruments, Houston, TX) (4). ECG traces were analyzed using LabChart 8 Pro (AD Instruments).

1. Rajaram MVS, Arnett E, Azad AK, Guirado E, Ni B, Gerberick AD, et al. M. tuberculosis-Initiated Human Mannose Receptor Signaling Regulates Macrophage Recognition and Vesicle Trafficking by FcRgamma-Chain, Grb2, and SHP-1. *Cell Rep.* 2017;21(1):126-40

**Supplemental Figures**

**
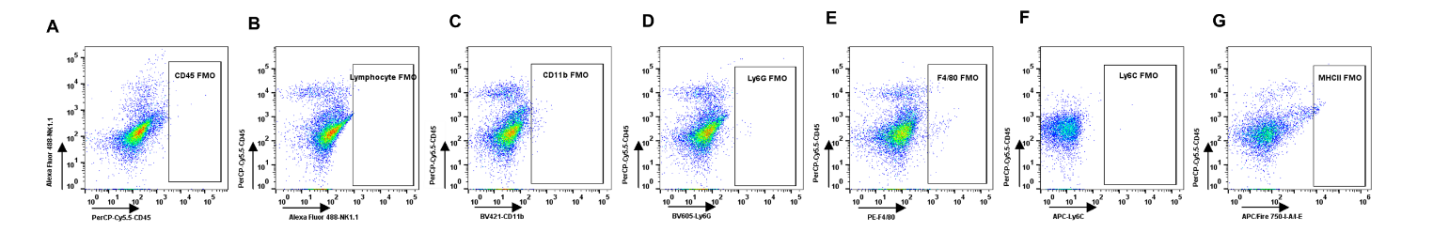
**

**Supplemental Figure 1.** **Use of FMO for gating control to manage autofluorescence in complex samples of Fig. 1.** (**A**) CD45 FMO, (**B**) Lymphocyte subsets FMO, (**C**) CD11b FMO (**D**) Ly6G FMO, (**E**) F4/80 FMO, and (**F**) Ly6C FMO.

**B**


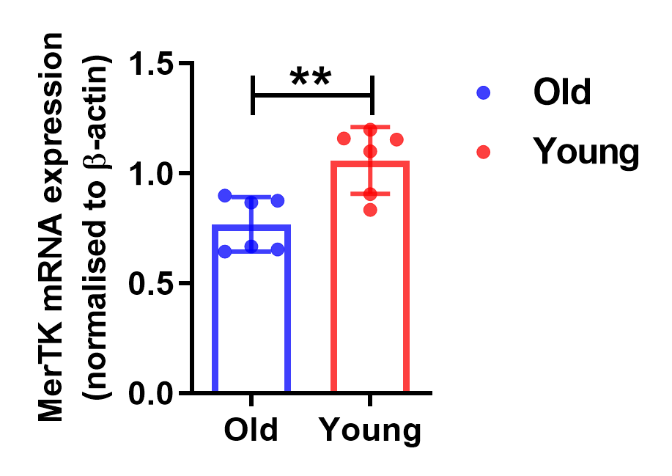


**A**


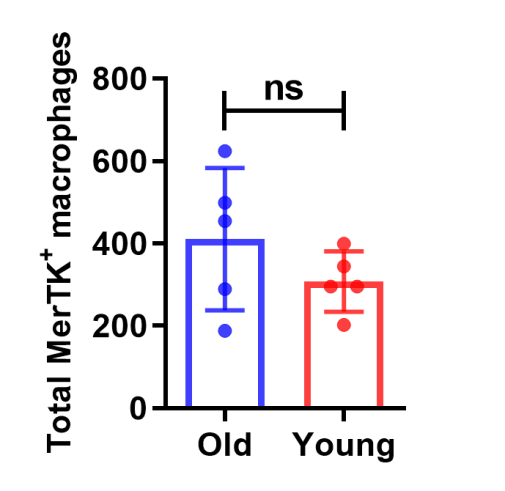


**Supplemental Figure 2: MerTK expression in the hearts of old and young mice.** The MerTK expression in the hearts of old and young mice was examined using both flow cytometry and qRT-PCR methods. Old (18 month) and young (6-8 weeks) C57BL/6 mice were euthanized, and hearts digested to obtain single cell suspensions. Cells were labelled with different myeloid cell markers and analyzed by flow cytometry for MerTK^+^ macrophages. Graph shown (**A**) is the total number of MerTK^+^ macrophages in the heart tissue from old and young mice. The single cell suspensions from the hearts of old and young mice were cultured in 12 wells plate and after 2 hrs incubation at 37°C CO_2_ incubator, the floating cells were washed with warm RPMI and the attached macrophages were lysed using Trizol reagent and RNA were obtained for analysis of MerTK expression. (**B**) Expression of MerTK mRNA determined by qRT-PCR with normalization to β-actin. Data shown in graphs is a representative data from three different independent experiments (4 mice per group). A two-tailed Student's t test was used to analyze the data (*p<0.05 and ** p<0.005).


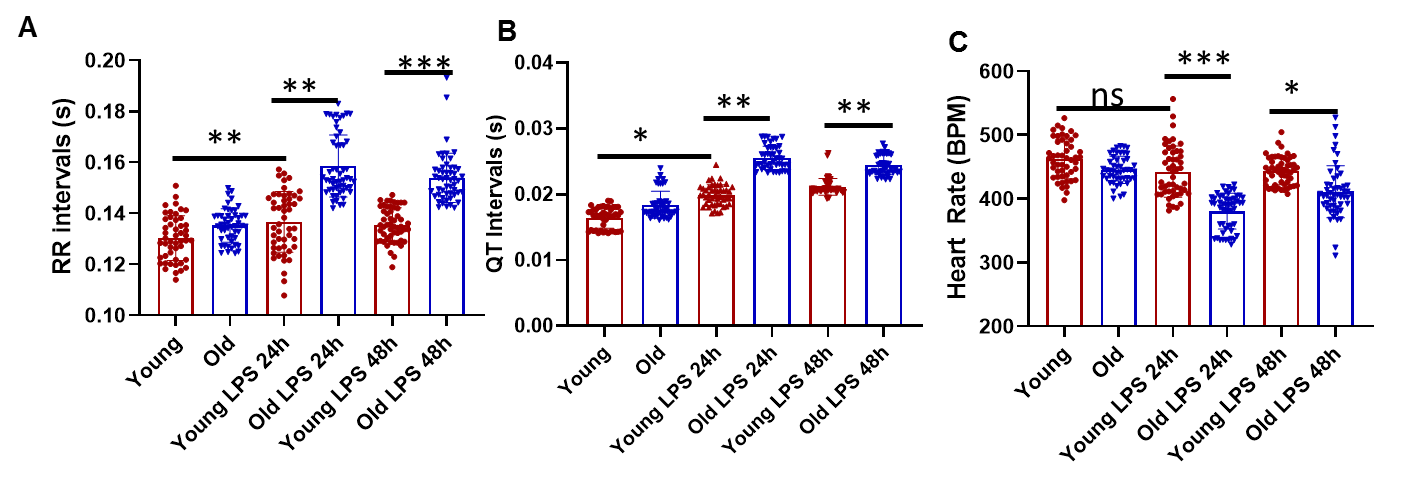


**Supplemental Figure 3. LPS infusion causes cardiac electrical dysfunction in both young and old mice.** Surface electrocardiogram (ECG) recordings of control and LPS injected (2 mg/kg) young and old mice at baseline and at 24 h and 48 h post treatment were acquired by Powerlab 4/30 (AD Instruments). ECG traces were analyzed using LabChart 8 Pro (AD Instruments). Shown are representative graph of RR intervals (**A**), QT intervals (**B**) and heart rate (**C**). Data shown are representative of 5 mice (N=5). A one-way ANOVA followed by the Bonferroni test was used to analyze the data (*p<0.05; ** p<0.005 and ***p<0.0005)


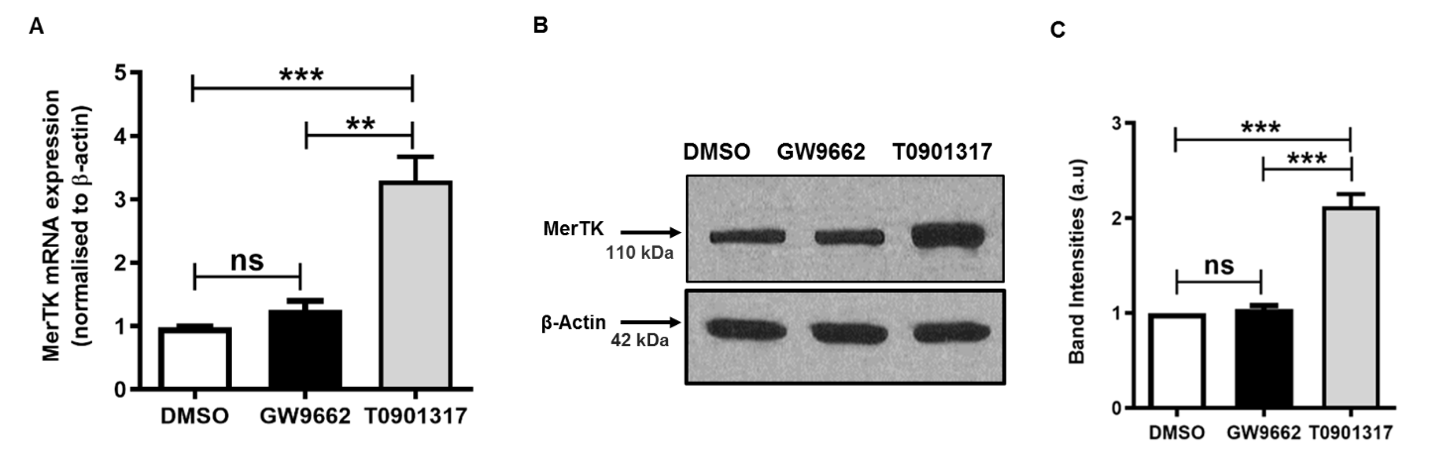


**Supplemental Figure 4. LXR agonist treatment induces the MerTK expression on the surface of macrophages in vitro.** BMDMs from young C57BL6 mice were treated with DMSO, PPRA-γ antagonist (GW9662) and LXR agonist (T0901317) for 24 h. RNA and whole cell lysates were obtained for analysis of MerTK expression. (**A**), Expression of MerTK mRNA determined by qRT-PCR with normalization to β-actin and expressed as fold change relative to DMSO control. (**B**), Western blot analysis of MerTK using antibody specific for MerTK and β-actin as loading control. (**C**), Band intensities of MerTK protein expression induction by LXR agonist (T0901317) in BMDMs. The data shown in the graphs are cumulative from three different independent experiments (N=3). A one-way ANOVA followed by the Bonferroni test was used to analyze the data (** p<0.005 and *** p<0.001).


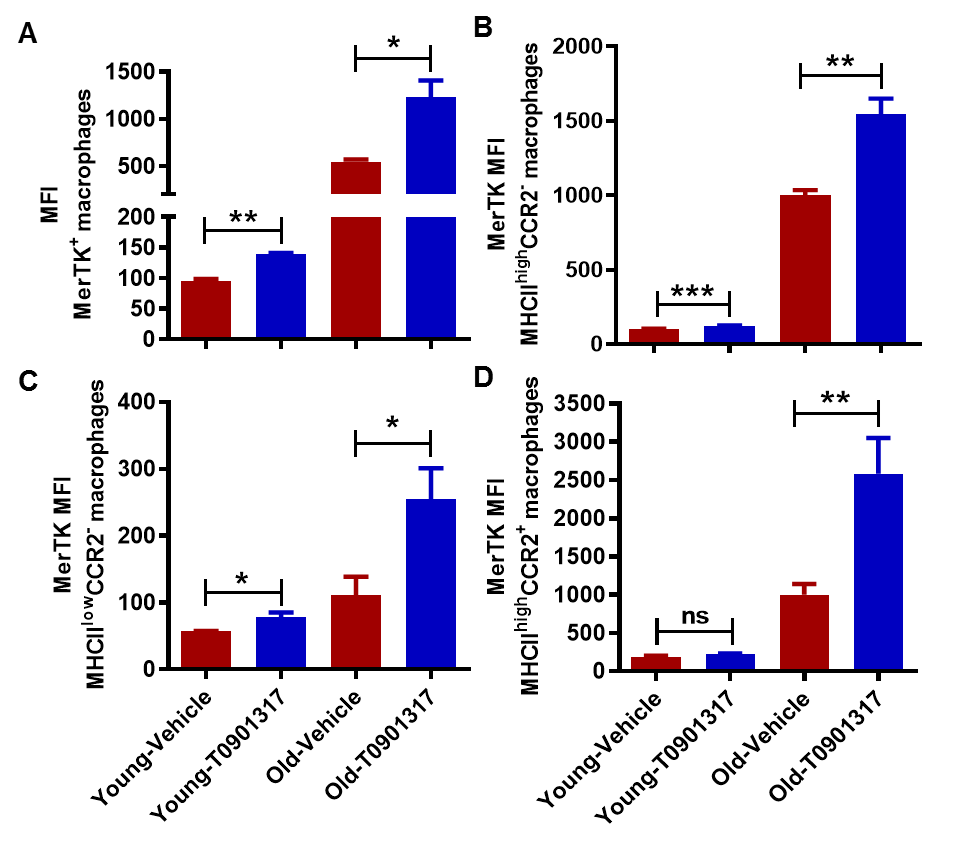


**Supplemental Figure 5: LXR agonist treatment enhances the number of MerTK^+^ CRMS in the hearts of young and old mice.** Young and old female C57BL/6 mice were intraperitoneal injected daily either with T0901317 (50mg/kg/day) or Vehicle for 9 days. At day 10 mice were euthanized and single cell suspensions were analyzed by flow cytometry. Graphs shown in (**A-D)** are MFI of MerTK in different CRM subsets. Data shown are accumulative data from 2 experiments with 3 mice/group. A one-way ANOVA followed by the Bonferroni test was used to analyze the data (*p<0.05; ** p<0.005 and ***p<0.0005).


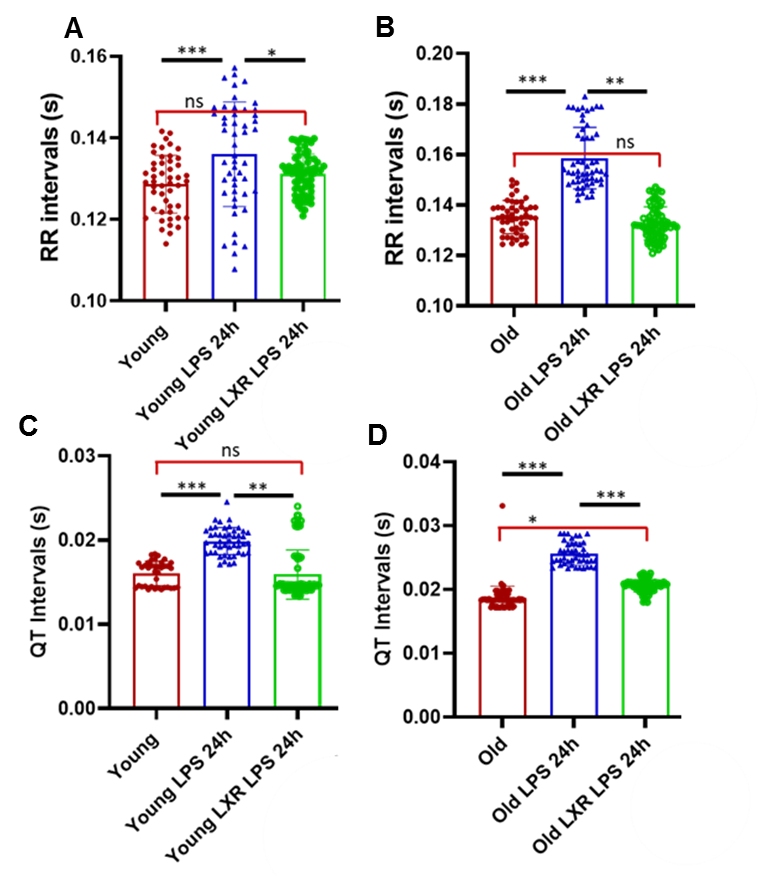


**Supplemental Figure 6: LXR agonist treatment reverses the LPS induced cardiac electrical function.** Young and old female C57BL/6 mice were intraperitoneal injected daily either with T0901317 (50 mg/kg/day) or Vehicle for 9 days. At day 10 mice were infused with LPS injection (2.5 mg/kg) or saline infused and surface electrocardiogram (ECG) recordings were obtained at baseline and at 24h post treatment by Powerlab 4/30 (AD Instruments). ECG traces were analyzed using LabChart 8 Pro (AD Instruments). Shown are representative graphs of RR intervals in individual young ((**A**) and old (**B**) mice, QT intervals of young (**C**) and old (**D**) mice. Data shown are representative of 3 experiments with 5 mice/group. A one-way ANOVA followed by the Bonferroni test was used to analyze the data (*p<0.05; ** p<0.005 and ***p<0.0005).
